# Continuous proline catalysis via leaching of solid proline

**DOI:** 10.3762/bjoc.7.197

**Published:** 2011-12-14

**Authors:** Suzanne M Opalka, Ashley R Longstreet, D Tyler McQuade

**Affiliations:** 1 Department of Chemistry and Chemical Biology, Cornell University, Ithaca, NY 14853; 2 Department of Chemistry and Biochemistry, Florida State University, Tallahassee, FL 32306

**Keywords:** aminoxylation, flow chemistry, heterogeneous catalysis, packed-bed microreactor, proline/thiourea catalysis

## Abstract

Herein, we demonstrate that a homogeneous catalyst can be prepared continuously via reaction with a packed-bed of a catalyst precursor. Specifically, we perform continuous proline catalyzed α-aminoxylations using a packed-bed of L-proline. The system relies on a multistep sequence in which an aldehyde and thiourea additive are passed through a column of solid proline, presumably forming a soluble oxazolidinone intermediate. This transports a catalytic amount of proline from the packed-bed into the reactor coil for subsequent combination with a solution of nitrosobenzene, affording the desired optically active α-aminooxy alcohol after reduction. To our knowledge, this is the first example in which a homogeneous catalyst is produced continuously using a packed-bed. We predict that the method will not only be useful for other L-proline catalyzed reactions, but we also foresee that it could be used to produce other catalytic species in flow.

## Introduction

**Figure 1:**
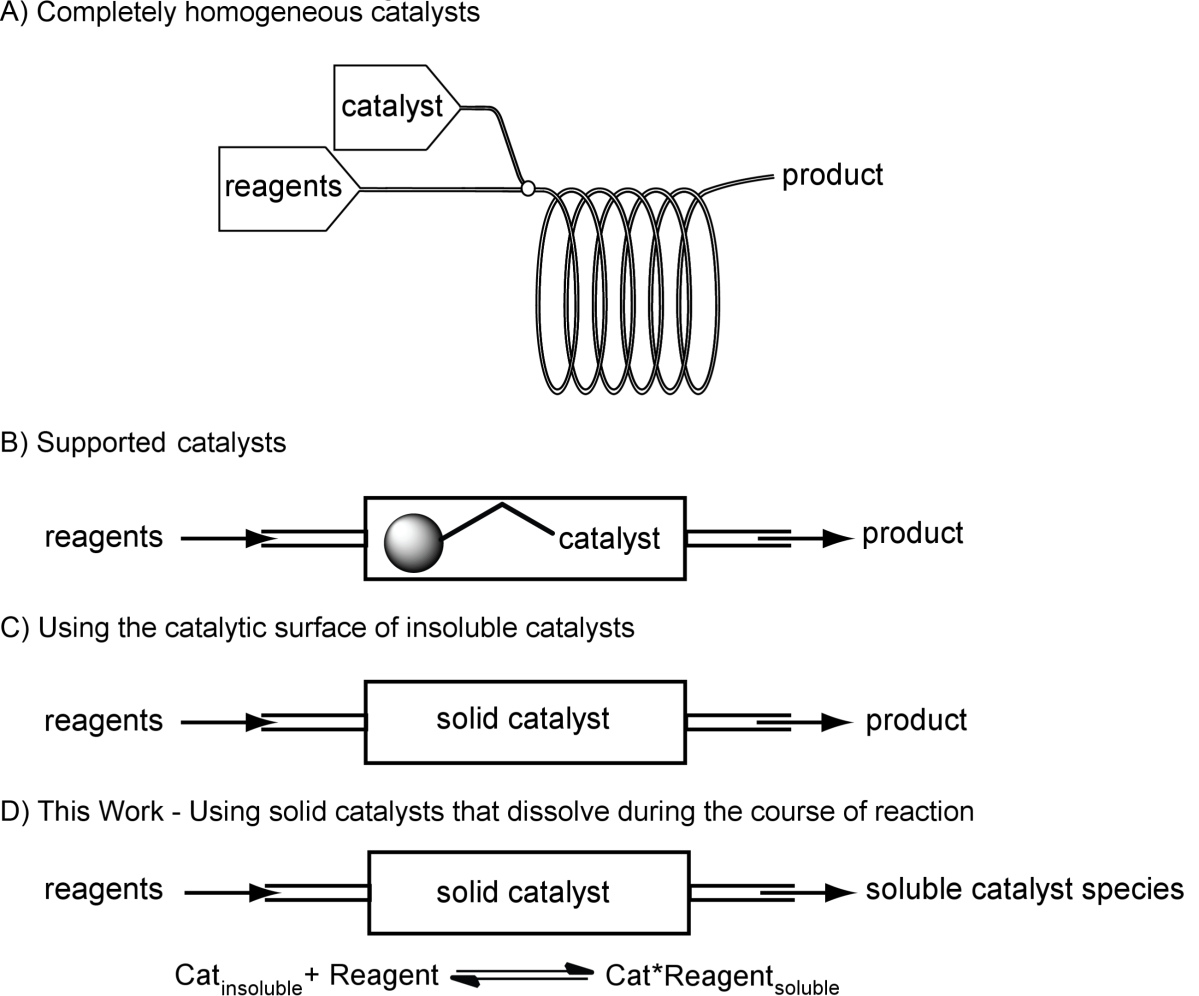
Methods for catalyst use in flow.

Continuous flow chemistry [1–3], performed in small dimension tubing or channels, differs from batch chemistry in that mixing and heat transfer are significantly faster and can be precisely controlled. In addition, continuous technology enables the generation and immediate use of unstable or hazardous intermediates [4–9] as well as the combination of many reactions in series to achieve multistep synthesis [9–13]. Despite the many favorable attributes of micro- and mesoflow reactors, the continuous use of solids remains challenging. The introduction of solids to a flow reactor is particularly difficult as most pumps function poorly with even small particulates, which in turn can result in channel clogging. Although the use of solids in flow has been the topic of a number of recent papers, they have focused on overcoming the challenges associated with the introduction and suspension of solid reagents and starting materials [14–18]. An area that has received less attention is the continuous use of solid catalysts (and catalyst precursors) that only partially or slowly dissolve into or react with the solution. (See Figure 1 for a comparison of solid catalysts that are used in flow.) Proline is an example of such a catalyst [19] (others include zero-valent transition metals, many solid acid catalysts, and other secondary amine catalysts). Proline is often added to a reaction mixture as a solid, and only a few mole percent dissolves into solution at any given time. Since proline is fairly inexpensive, it is an attractive test catalyst for the design of new methods to utilize solid catalysts or catalyst precursors in flow.

Current strategies involving the use of catalysts with limited solubility in flow rely on them being supported on resins or polymers (Figure 1A and B). This can be an attractive method as the catalysts are often easily recycled [20–24]. Finding a suitable solid support for a reaction, however, can prove time-consuming and expensive. In addition, care must be taken to identify a support that provides both high activity for the catalyst and appropriate swelling properties to enable adequate mass transport (often the best solvent for the resin will not be the best for the reaction) [25–29]. With researchers becoming increasingly interested in developing continuous-flow processes, the rapid assessment of catalyst conditions necessary for potential synthetic routes requires a simple approach to deal with limited-solubility catalysts.

We have both a long-standing interest in the production, use and management of solids [30–33] and reactions [34–35] in flow as well as in proline catalysis [36–37]. This prompted us to consider new strategies for the implementation of proline in a continuous-flow system without resorting to proline analogues or tethered catalysts [38–40]. Achieving this goal would enable us and others to perform proline-catalyzed reactions, aldol [41–43] and Mannich [42] reactions as well as α-functionalizations (α-aminoxylation, α-amination or α-halogenations), continuously [44].

We hypothesized that the proline-catalyzed α-aminoxylation could be implemented in flow with reasonably short residence times using a urea additive. Many researchers, including us [36–37], have found that urea [45] additives increase the rate of various proline-catalyzed reactions [46–50]. The role that ureas play remains unclear, and a number of hypotheses have been suggested. Initially, researchers gathered ^1^H NMR, UV, and fluorescence data to show that ureas enhance the solubility of proline through a host–guest interaction between the urea and proline carboxylate: A substrate-independent model [49–50]. However, it has been proposed that substrate–urea–proline interactions may also contribute to the rate enhancement [50]. Our group observed that a urea tethered to a tertiary amine increases the rate of a number of batch reactions, including the α-aminoxylation reaction [36–37]. For the α-aminoxylation reaction, we proposed that the urea promotes formation of the active enamine intermediate through breakdown of the putative oxazolidinone intermediate: A substrate-dependent model. Here, we report that a packed-bed of solid proline can be used to create a homogeneous catalyst, and we use this system to perform continuous α-aminoxylations. Not only do we illustrate a unique use of catalysts in flow, but we provide additional insight into the role of additives in proline-catalyzed reactions.

## Results and Discussion

**Scheme 1:**
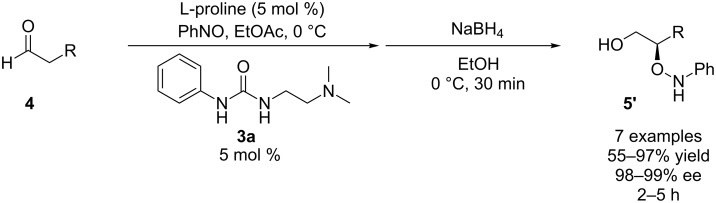
Prior results for batch α-aminoxylation reaction.

In our previously published batch work, we found that the combination of L-proline and bifunctional urea **3a** greatly accelerated the rate of α-aminoxylation (Scheme 1). It was shown that a longer linker between the urea and amine functionality enhanced the rate of reaction (see Supporting Information of [37]). The rate enhancement enabled the reaction to be performed in greener solvents such as ethyl acetate instead of the more commonly used chloroform. We have a long standing interest in the conversion of the reaction into a continuous process, but recognized that the solubility of proline would hinder its use in flow. To circumvent this problem, we envisioned using a cartridge of solid proline as a precatalyst source, whereby the flow of a combination of solvents, reactants and cocatalysts through the packed-bed would produce the active, homogeneous, oxazolidinone catalyst.

**Figure 2:**
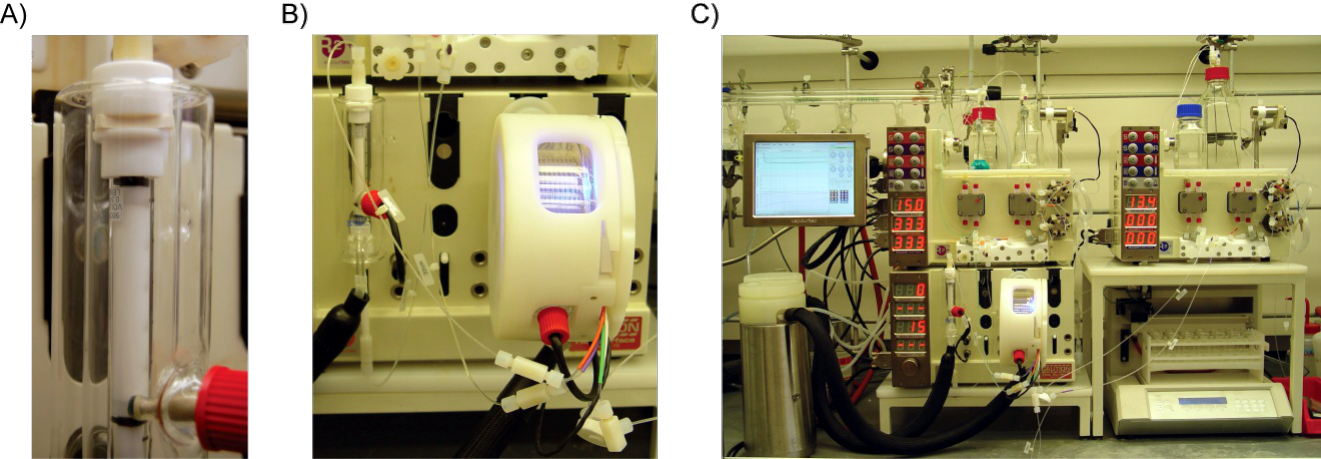
General reactor setup. A) A glass Omnifit column is packed with 1 g of proline. B) The column is then placed in-line with a 10 mL PFA coil-tube reactor. C) The components are connected to HPLC pumps for solvent and reagent inputs. The reactor is controlled by a computer in order to program the timing of the reagent and solvent inputs and fraction collection.

To test our hypothesis, we used a Vapourtec R series reactor system [51] consisting of HPLC pumps for solvent and reagent inputs, a low-temperature tube reactor containing a glass column packed with 1 g of proline, and a low-temperature 10 mL PFA coil-tube reactor in which each reagent stream could be precooled prior to mixing (Figure 2). As we demonstrate below, the success of our experiments depended on the ability of the system to heat, or cool, the packed-bed and the reaction coil independently of one another.

Using this device configuration, experiments were performed to identify the conditions that favor the reaction between the aldehyde and the proline packed-bed in order to yield enough soluble oxazolidinone catalyst to support rapid α-aminoxylations. We were particularly interested to determine which substrate and additive components were necessary to dissolve the solid proline. Since the inherent solubility of proline in ethyl acetate is very low, we extrapolated that this solvent alone would be insufficient to dissolve enough catalytic proline [37,52]. Furthermore, we knew from our previous batch work that a urea additive would be beneficial to provide reaction rates suitable for use in flow [37].

Therefore, various combinations of reagents and catalysts entering the packed proline column were investigated. For our initial experiments we selected a 15 min coil residence time and temperature of 0 °C for both the column and the coil, based on our prior knowledge of the reaction in batch. We began by determining the necessity of a urea cocatalyst. We were surprised to find that when hexanal alone was passed through the column and combined with nitrosobenzene in the coil, the desired product was not detected by crude ^1^H NMR analysis (Table 1, entry 1). This indicates that, with this reactor setup, the reaction is too slow without a urea additive to be a viable method. Additionally, flowing thiourea **3b** (0.047 M in EtOAc) through the proline packed-bed prior to combination with the other reaction partners resulted in no detectable reaction (Table 1, entry 2). This shows that thiourea **3b** alone cannot solubilize enough proline to support the reaction. When hexanal alone, however, was passed through the column and combined with the remaining reagents in the coil (including thiourea **3b**) the reaction produced 27% yield and 99% ee (Table 1, entry 3). This indicates that the aldehyde alone can react with solid proline to produce a reactive homogeneous catalyst. However, when both thiourea **3b** and hexanal were used in the same stock solution and passed through the column the reaction attained 43% yield with 98% ee (Table 1, entry 4). This increase in yield, relative to when hexanal alone was passed through the column, suggests that the rate of proline leaching is enhanced by the addition of thiourea **3b**. Consequently, it appears that our observed rate enhancements with thiourea **3b** cannot be attributed to a model involving only urea–proline interactions resulting in enhanced solubility, and that substrate–urea–proline interactions are responsible for the observed reactivity when using this combination of thiourea, substrate and proline.

**Table 1:** Screening of the reactor setup.

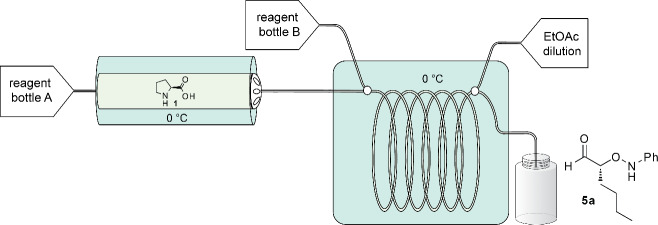				

entry	reagent bottle A	reagent bottle B^a^	yield (%)	ee (%)^b^

1	hexanal	nitrosobenzene	nr^c^	na
2	thiourea **3b**	nitrosobenzene + hexanal	nr^c^	na
3	hexanal	nitrosobenzene + thiourea **3b**	27^d^	99
4	hexanal + thiourea **3b**	nitrosobenzene	43^d^	98

^a^Entry 4 also contained dodecane as an internal standard. ^b^Determined by chiral HPLC. ^c^nr = no reaction as determined by ^1^H NMR analysis of the crude reaction mixture after reduction. ^d^Isolated yield (due to instability of the aldehyde, products were reduced to their corresponding 2-aminooxy alcohols in batch, prior to isolation). na = not applicable. See Supporting Information File 1 for detailed reaction conditions.

We were delighted to find that further increasing the residence time of the coil to 20 min with the same reagent configuration resulted in 69% yield (Table 2, entry 2). For further experiments, we therefore used a setup in which a thiourea/aldehyde stock solution was passed through the proline packed-bed before entering the coil and reacting with nitrosobenzene.

**Table 2:** Screening of temperature and residence time.

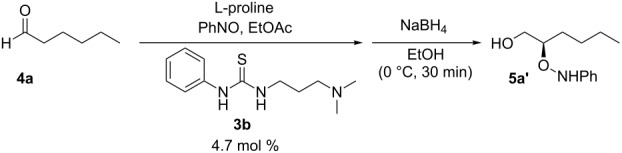					

entry	column temperature (°C)	coil temperature (°C)	residence time (min)	yield (%)^a^	ee (%)^b^

1	0	0	15	43	98
2	0	0	20	69	98
3	0	15	15	82	98
4	0	15	20	85	98
5	0	15	10	61	98
6	0	10	20	84	98
7	0	5	20	86	98
8	0	5	25	81	98
9	−5	5	20	84	98
10	5	5	20	75	98
11	10	5	20	66	98
12	20	5	20	68	98

^a^Isolated yield (due to instability of the aldehyde, products were reduced to their corresponding 2-aminooxy alcohols in batch, prior to isolation). ^b^Determined by chiral HPLC. See Supporting Information File 1 for detailed reaction conditions.

**Figure 3:**
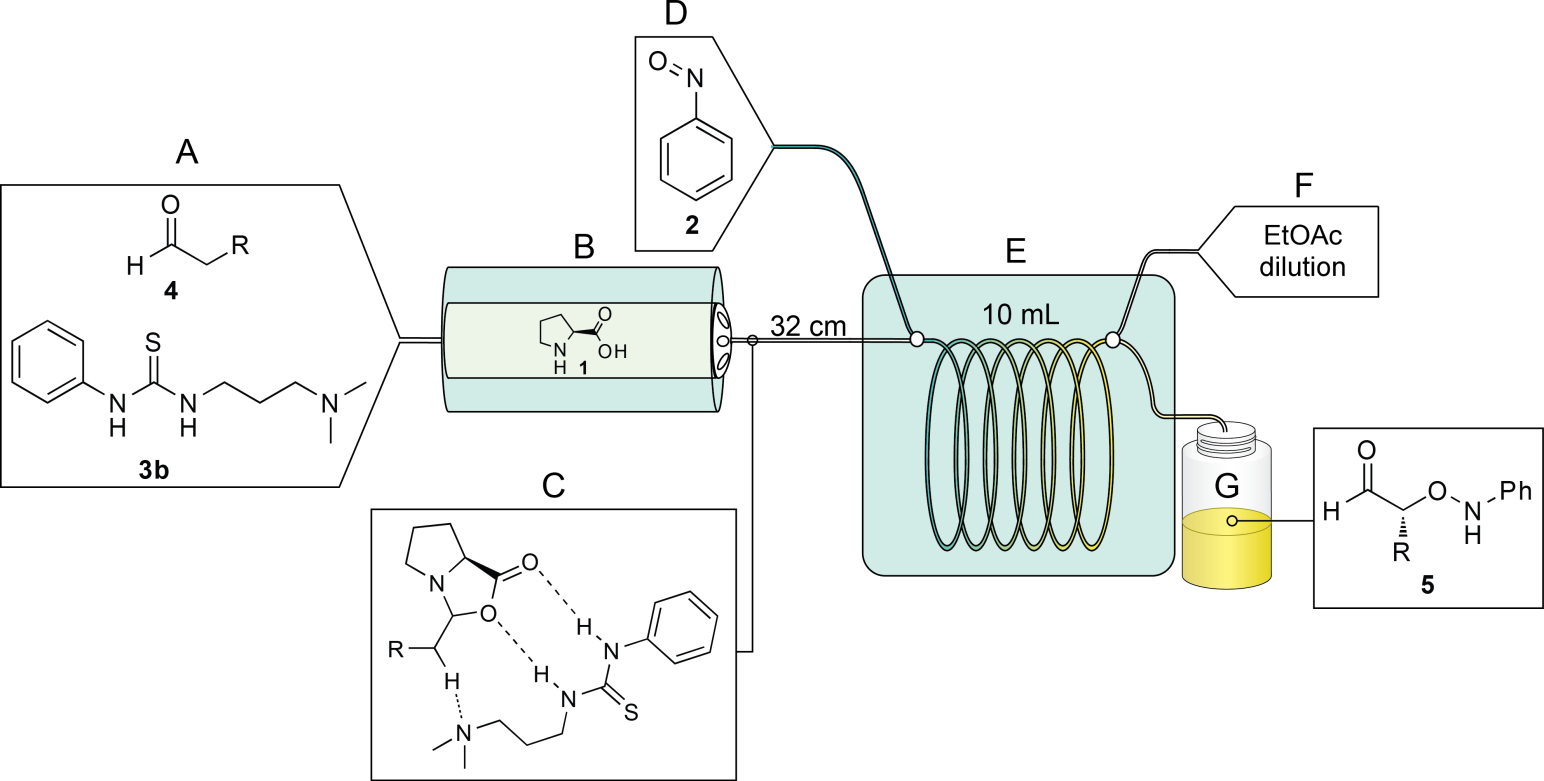
Schematic of the reactor setup. As the starting aldehyde and thiourea **3b** (A) enter the proline packed-bed (B) an oxazolidinone intermediate is formed, drawing the proline into the solution (C). Upon precooling in a reactor coil (E) the intermediate is mixed with nitrosobenzene (D). Prior to exiting, ethyl acetate is added to dilute the reaction (F) and product is collected into vials (G) for further reduction, work-up, and isolation.

As all of the reactions performed in Table 1 had the same residence time and temperature, the yield can be used as a rough proxy for reaction rate. We conjecture, based on our prior work in this area, that the aldehyde slowly reacts with solid L-proline in the cartridge to form the soluble oxazolidinone intermediate (Figure 3, part C), leaching proline out of the column and into the coil for reaction with nitrosobenzene. The increased yield observed when both hexanal and thiourea **3b** were passed through the proline-bed suggests that more catalyst was drawn into the solution, resulting in a faster reaction rate. In our prior batch experiments, we proposed that the urea aided the breakdown of the oxazolidinone intermediate (Figure 3, part C) for rapid reaction with nitrosobenzene, and this thesis is supported by our observations reported herein.

With evidence for adequate proline transport into the coil, optimization experiments were performed. Based on previously published studies on the α-aminoxylation, we believed that careful control of the temperature would be necessary to avoid the formation of byproducts and to realize high enantioselectivity. The forced convection cooling system facilitated easy and precise temperature control of both the column and coil independently. Reported byproducts include the self-aldol product, the formation of azoxybenzene from the reaction of the desired product with nitrosobenzene, and finally azobenzene by product disproportionation [53–55]. Byproduct suppression is both solvent and temperature dependent. Hayashi reported that when the reaction is performed at room temperature in acetonitrile with 30 mol % proline, the reaction is complete in 10 min, but achieves only 29% yield [55]. MacMillan, however, found the reaction to be rapid in chloroform at room temperature with 78% yield, using 10 mol % proline [56]. In addition, our prior batch work with urea **3a** in ethyl acetate found that the α-aminoxylation of hexanal worked well at 0 °C with 5 mol % proline in 2 h. Therefore, we studied the impact of both the packed-bed and reaction-coil temperature on the enantioselectivity and product yield.

To begin with, we kept the column temperature at 0 °C and increased the *coil* temperature to 15 °C. Under these conditions a 15 min residence time provided 82% yield (Table 2, entry 3). Increasing the residence time to 20 min provided little gain in yield, while reducing the residence time to 10 min afforded only 61% yield (Table 2, entries 4 and 5). We found that as the coil temperature was decreased from 15 to 10 and then to 5 °C the yield corresponding to a 20 min residence time remained steady (Table 2, entries 4, 6, and 7). A further reduction to 0 °C, however, showed a decrease to 69% (Table 2, entry 2). At each of these temperatures the enantioselectivity remained high.

Next, the *packed-bed* temperature was varied to determine how temperature influenced the formation of the active catalyst species from hexanal, proline, and thiourea **3b**. We found that at column temperatures less than 0 °C the reaction performed well (Table 2, entries 7 and 9). As the temperature was increased to 5, 10, and 20 °C the yield dropped and was 68% at 20 °C (Table 2, entries 10, 11, and 12). Therefore, for further experiments we chose a column temperature of 0 °C and a coil temperature of 5 °C with a 20 min residence time. It is clear, however, from the parameters investigated, that when simple sterically unencumbered aldehydes are used this reaction works well under a variety of conditions.

**Figure 4:**
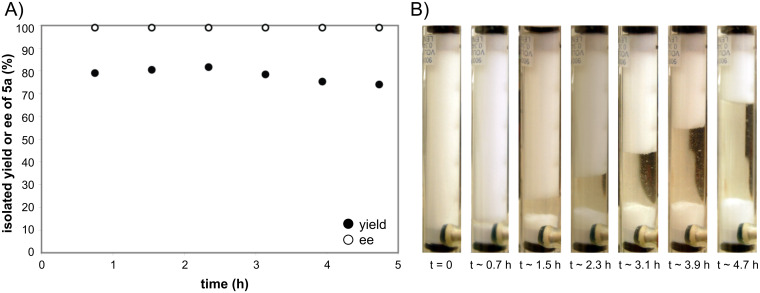
The long-term stability of a proline packed bed in the α-aminoxylation reaction of hexanal. A solution of hexanal (3 M in EtOAc) and thiourea **3b** (0.047 M in EtOAc) was passed through a packed-bed of proline (entering at the bottom of the column and exiting at the top) at 0 °C combined with a solution of nitrosobenzene (1 M in EtOAc) in a coil at 5 °C with a 20 min residence time in the coil, for over 4 h. A) 20 mL of product was periodically collected into vials, reduced in batch, and purified. The resulting yields and enantioselectivities were plotted as a function of time. B) Close up images of the proline column (see Figure 2A) showing the amount of proline consumed during the course of the reaction.

To assess the long term stability and activity of the L-proline packed-bed, the system was run continuously for over 4 h. After the system reached equilibrium, 20 mL fractions of product were periodically collected, reduced and purified. The data shown in Figure 4 indicate that the reaction is stable over this period of use. During the ~5 h collection period, assuming an average yield of 78%, approximately 9.8 g was produced. Over the entire run 80 mL of hexanal/thiourea **3b** stock solution was passed through the column. Upon completion of this study it was determined that 82% of the proline was consumed (823.1 mg out of 1 g) (Figure 4).

**Scheme 2:**
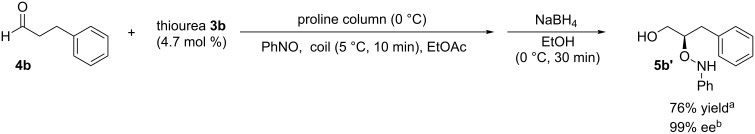
Reaction with 3-phenylpropionaldehyde through reactor setup. ^a^Isolated yield, due to the instability of the aldehyde, the product was reduced in batch to the corresponding 2-aminoxy alcohol prior to isolation. ^b^Determined by chiral HPLC.

As further support that the direct use of solid catalysts in flow is a viable strategy, two additional substrates, 3-phenylpropionaldehyde and isovaleraldehyde, were selected because they have different properties compared to hexanal, and thus we predicted that they would require alterations to the system to maximize yield. As a starting point, the conditions optimized for hexanal were investigated. With 3-phenylpropionaldehyde, the use of a 0 °C column temperature, 5 °C coil temperature and a 20 min coil residence time led to rapid reaction (based upon color change in the coil) and subsequent reactor clogging. This led us to conclude that this aldehyde reacts rapidly with proline to yield an oxazolidinone with lower solubility than hexanal and that lowering the overall residence time would limit the amount of aldehyde reacting with proline. We confirmed that our assertion was reasonable by reducing the residence time to 10 min to provide the product in 76% yield and 99% ee (Scheme 2).

**Scheme 3:**
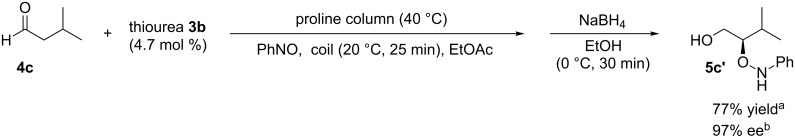
Reaction with isovaleraldehyde through reactor setup. ^a^Isolated yield, due to the instability of the aldehyde, the product was reduced in batch to the corresponding 2-aminooxy alcohol prior to isolation. ^b^Determined by chiral HPLC.

When isovaleraldehyde was investigated under the optimized hexanal conditions, i.e., 0 °C column temperature, 5 °C coil temperature and a 20 min coil residence time, there was little conversion as judged by GC analysis. We were not surprised by this observation, because increased steric hindrance about the aldehyde can suppress the rate of oxazolidinone formation. With limited proline (in the form of oxazolidinone) entering the system, the rate of α-aminoxylation decreases significantly. From our hexanal and 3-phenylpropionaldehyde experiments, we learned that by adjusting one of three parameters (residence time or the coil or packed-bed temperature) we could improve the amount of catalyst entering the system. In this particular case, we predicted that, unlike 3-phenylpropionaldehyde, the isovaleraldehyde would form the oxazolidinone slowly. Furthermore, we predicted that raising the packed-bed temperature would increase the rate of proline/isovaleraldehyde reaction resulting in more rapid formation of the soluble catalyst species. A quick survey of temperatures revealed that a 40 °C packed-bed temperature and a 20 °C coil temperature with a 25 min residence time provided 76% yield and 97% ee (Scheme 3). It is apparent from these results and our initial conditions that substrate-to-substrate optimization can be rapidly achieved by a quick survey of the three critical parameters. The data further highlight the value of running reactions continuously.

## Conclusion

We have demonstrated that a packed-bed of proline can be used to continuously form a soluble catalyst through reaction with an aldehyde and cocatalytic urea. The formed soluble catalyst can support a variety of α-aminoxylation reactions with good yields and high enantioselectivities. The system is designed so that the first step involves the flow of aldehyde and urea solution through the proline packed-bed to generate the catalytic intermediate (presumably an oxazolidinone). This catalyst solution is then combined with a stream of nitrosobenenze, resulting in the α-aminoxylation. The most critical parameters that control the yield and selectivity were identified, and these parameters were varied in order to optimize the system for each substrate. We predict that this basic setup can be adapted to generate a wide range of other catalysts by replacing proline with another solid catalyst precursor. We are currently investigating the combination of ligands and solid metal salts to generate transition-metal catalysts continuously.

## Supporting Information

The Experimental Section describes reactor setup and operational details, screening conditions, synthesis, purification and characterization data of all catalysts, and the starting materials and substances given in this article.

File 1 Experimental Section.
