# Supplementary material for: Continuous proline catalysis via leaching of solid proline
Source: Beilstein J Org Chem. 2011 Dec 14;7:1671–9. doi: 10.3762/bjoc.7.197 (PMC3252872; doi:10.3762/bjoc.7.197)

Supporting Information

for

Continuous proline catalysis via leaching of solid proline

Suzanne M. Opalka[1], Ashley R. Longstreet[2] and D. Tyler McQuade*[2]

Address: [1]Department of Chemistry and Chemical Biology, Cornell University, Ithaca, NY 14853 and [2]Department of Chemistry and Biochemistry, Florida State University, Tallahassee, FL 32306

Email: D. Tyler McQuade - mcquade@chem.fsu.edu

* Corresponding author

Experimental Section

Contents	Page
1. General Information	S2
2. Catalyst and Reactant Synthesis	S2–S3
3. General Reactor Setup	S3–S4
4. Experiments to Assess Reactor Setup	S4–S5
5. General Screening Procedures	S5–S6
6. Assessing Long Term Stability and Activity of the Proline Packed Bed	S6
7. Representative Pump Trace	S7
8. Product Characterization	S7–S8
9. References	S8
10. Chromatograms of Racemic and Enantiomerically Enriched Products	S9–S11
11. ^{1}H and ^{13}C NMR Spectra of Chemical Compounds	S12–S23

1. General Information

Flash chromatography was performed on silica gel (230–400 mesh). For analytical thin layer chromatography (TLC), silica gel 60 F_{254} plates were used. All commercial reagents were used without further purification, with the following exceptions: Hexanal, hydrocinnamaldehyde, and isovaleraldehyde were distilled prior to use. We found it helpful to filter the stock solutions of aldehyde, thiourea, and nitrosobenzene through fritted syringes and ethyl acetate with standard filter paper prior to use, as this removed any fine particulates. Proton nuclear magnetic resonance (^{1}H NMR) spectra and carbon nuclear magnetic resonance (^{13}C NMR) spectra were recorded on 600 MHz spectrometer. Chemical shifts for protons are reported in parts per million downfield from tetramethylsilane or referenced to residual solvent. Chemical shifts for carbon are reported in parts per million downfield from tetramethylsilane or referenced to residual solvent. Data are represented as follows: chemical shift, multiplicity (br = broad, s = singlet, d = doublet, t = triplet, q = quartet, m = multiplet), coupling constants in Hertz (Hz), integration.

Reactions were performed with a commercially available Vapourtec R series reactor controlled by FlowCommander™ software. Solid proline catalyst was packed into a glass Omnifit column (6.6 mm diameter) fitted with Vapourtec end caps and sintered glass frits. All tubing and fittings were supplied with the reactor, but the tubing was standard 1.00 mm bore PFA and standard PTFE fittings.

2. Catalyst and Reactant Synthesis

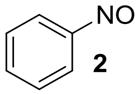

2

Nitrosobenzene (2). Prepared according to previously reported method with some modifications [1]. A solution of sodium tungstate dihydrate (1.02 g, 3.08 mmol, 0.014 equiv) in 20 mL hydrogen peroxide (30 wt. % in H_2O) was cooled to 15–19 ° C. Aniline (20 mL, 220 mmol, 1 equiv) was added at a constant flow rate of 0.333 mL/min by syringe pump and the temperature was kept between 15–19 °C. Throughout the course of the reaction, three 5 mL portions of hydrogen peroxide were added. From the start of the aniline addition, the reaction was stirred for 1.25 h. The resulting solid was collected by vacuum filtration then stirred in 150 mL of water for 10 min to solubilize any residual catalyst. The solid was collected by vacuum filtration, rinsed with water and then cold ethanol. The solid was recrystallized from ethanol (0.1 g of solid to 1 mL of ethanol). The resulting crystals were collected and dried under vacuum over P_2O_5 for 24 h to yield **2** (8.86 g, 38%) as white crystals with some residual water. ^{1}H NMR: (600 MHz, $CDCl_3$) δ 7.90 (d, J = 7.3 Hz, 2H), 7.71 (m, 1H), 7.62 (t, J = 7.8 Hz, 2H) ppm. ^{13}C NMR (151 MHz, $CDCl_3$) δ 166.1, 135.7, 129.5 (2C), 121.1 (2C) ppm.

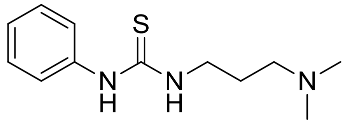

3b

1-(3-(dimethylamino)propyl)-3-phenylthiourea (3b). Phenyl isothiocyanate (5.0 mL, 41.9 mmol, 1 equiv) and 3-(dimethylamino)-1-propylamine (5.8 mL, 46.0 mmol, 1.1 equiv) were added to 460 mL of ethyl acetate. The reaction was stirred at room temperature for 17 h and then concentrated in vacuo. The crude product was then recrystallized in 35% EtOAc/hexanes and washed with cold hexanes to yield **3b** (9.05 g, 91%) as white crystals. ^{1}H NMR: (600 MHz, CD_3OD) δ 7.39 (t, 2H, J = 7.8 Hz), 7.29 (d, 2H, J = 7.3 Hz), 7.23 (t, 1H, J = 7.4 Hz), 3.62 (br s, 2H), 2.35 (t, 2H, J = 6.9 Hz), 2.11 (s, 6H), 1.75 (m, 2H, J = 6.9 Hz) ppm. ^{13}C NMR (150 MHz, CD_3OD) δ 182.1, 139.5, 130.6 (2C), 127.2, 126.3 (2C), 59.0, 45.5 (3C), 27.3 ppm. Anal. Calcd. For $C_{12}H_{19}N_3S$ C: 60.72, H: 8.07, N: 17.70, S: 13.51. Found C: 60.86, H: 8.01, N: 17.64, S: 13.22.

3. General Reactor Setup

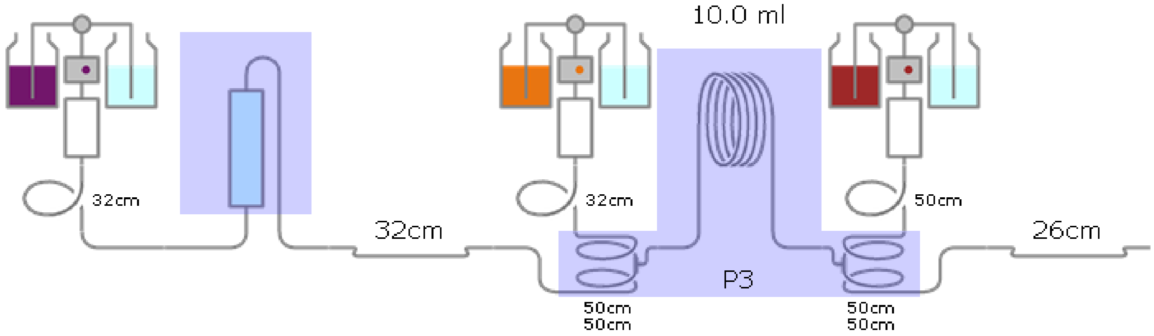

Description from left to right: The first pump was connected with 32 cm of drop tubing to a glass Omnifit column (6.6 mm diameter) containing 1.0 g of proline with a measured void volume of ~1.4 mL. An additional 32 cm of tubing connected the column to a 10.0 mL tube reactor were reagents were precooled prior to mixing (see below for dimensions). The second pump was also connected to an inlet of the tube reactor with 32 cm of drop tubing. The final pump(s), were connected to the "quench inlet" of the tube reactor with 50 cm of drop tubing. Depending on the residence time and total flow rate needed, 1–2 pumps were used, each with 50 cm of drop tubing. To accommodate two pumps, a t-mixer outside the tube reactor was used to generate a single stream of ethyl acetate. The dilution was used to slow the reaction before exiting the coil for collection in a fraction collector. Two 8 bar backpressure regulators (not shown) were connected in series along with additional tubing (not shown) to reach the fraction collector.

Detailed schematic of the 10.0 mL coil reactor

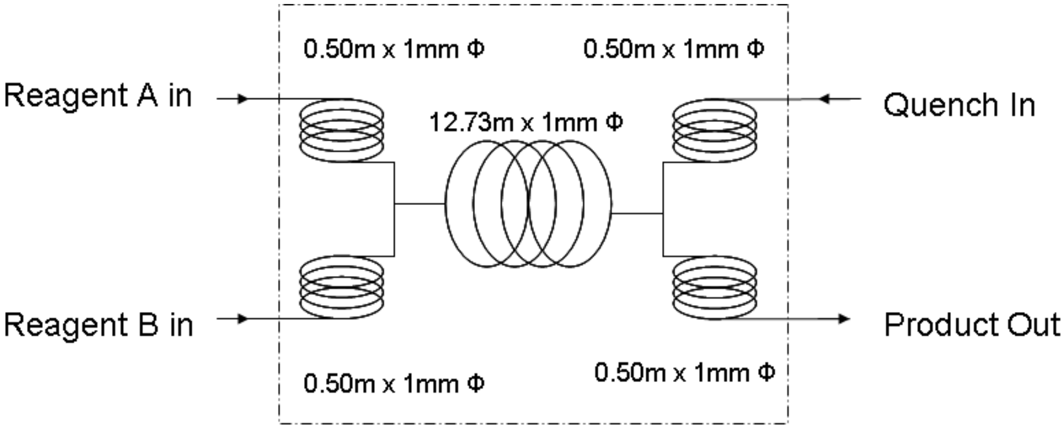

4. Experiments to assess reactor setup (Table 1)

A series of reactions were run to assess the best configuration for reactor operation. As a general setup, a glass Omnifit column (6.6 mm diameter) and a 10 mL reactor coil, each with independent temperature control were used. 1.0 g of solid L-proline was packed into the Omnifit column. The reactor was flushed with ethyl acetate prior to use. One stock solution was dispensed from pump A and the other stock solution was dispensed from pump B. Additional ethyl acetate was added by using pump C and D or just C depending on the total flow rate. The purpose of these experiments was to determine which reagents needed to flow through the packed bed of proline in order for the reaction to proceed efficiently. The following stock solutions were prepared.

Reaction 1 (Table 1, entry 1):
Reagents through column (pump A): 3 M hexanal solution in EtOAc
Reagents entering coil (pump B): 1 M nitrosobenzene
Volumetric ratios for pump A/pump B/ethyl acetate dilution → 1:1:29

Reaction 2 (Table 1, entry 2):
Reagents through column (pump A): 0.047 M **3b** solution in EtOAc
Reagents entering coil (pump B): 1 M nitrosobenzene, 3 M hexanal
Volumetric ratios for pump A/pump B/ethyl acetate dilution → 1:1:29

Reaction 3 (Table 1, entry 3):
Reagents through column (pump A): 3 M hexanal solution in EtOAc
Reagents entering coil (pump B): 1 M nitrosobenzene, 0.047 M **3b**
Volumetric ratios for pump A/pump B/ethyl acetate dilution → 1:1:29

Reaction 4 (Table 1, entry 4)
Reagents through column (pump A): 3 M hexanal and 0.047M **3b** solution in EtOAc
Reagents entering coil (pump B): 1 M nitrosobenzene, 0.1 M dodecane solution in EtOAc
Volumetric ratios for pump A/pump B/ethyl acetate dilution → 1:1:39

The following flow rates, depending on the volumetric ratios indicated above, were used for the pumps:

Residence time (minutes)	Pump A flow rate (mL/min)	Pump B flow rate (mL/min)	Pump C flow rate (mL/min) (1:1:29 ratio)	Pump C+D flow rate (mL/min) (1:1:39 ratio)
15	0.333	0.333	9.667	
15	0.333	0.333		13.0

For each reaction, the temperatures of both the column and the coil were set at 0 °C and a coil residence time of 15 minutes was used to assess each configuration. Once the system reached equilibrium, a fraction collector was used to collect diluted reaction mixtures (amount varied depending on dilution conditions) containing a theoretical product amount of 0.976 mmol. After collection, the samples were poured into a suspension of sodium borohydride (148 mg, 3.9 mmol, 4 equiv) in 10 mL of ethanol at 0 °C. The vials were rinsed with an additional 10 mL of ethanol and stirred for 30 min. 25 mL of saturated sodium bicarbonate was added and stirred, followed by 50 mL of water with further stirring. Any additional water was added to solubilize the precipitate prior to extraction. The reaction mixture was then transferred to a separatory funnel, the layers separated and the organic layer collected followed by extraction of the aqueous phase 2x with dichloromethane. The combined organic layers were dried over $MgSO_4$, filtered, and concentrated. The product was directly chromatographed with a gradient of 15% EtOAc/hexanes → 50% EtOAc/hexanes to afford the desired compound. The enantioselectivities were determined by chiral HPLC analysis.

5. General Screening Procedures (Table 2)

A stock solution of nitrosobenzene and dodecane (used as an internal standard) was prepared in ethyl acetate. A separate stock solution of thiourea **3b** and hexanal in ethyl acetate was also prepared. A glass Omnifit column (6.6 mm diameter) was packed with 1.0 g of L-proline. The reactor setup was flushed with ethyl acetate prior to use. One stock solution was dispensed from pump A and the other stock solution was dispensed from pump B. Additional ethyl acetate was added by using pumps C and D or just C depending on the total flow rate. For each experiment the following solutions were prepared and the reactor setup was programmed:

Reagents through column (pump A): 3 M hexanal and 0.047 M **3b** solution in EtOAc
Reagents entering coil (pump B): 1 M nitrosobenzene, 0.1 M dodecane solution in EtOAc (except Table 2, entry 2 did not contain dodecane)
Volumetric ratios for pump A/pump B/ethyl acetate dilution → 1:1:39

After the system reached equilibrium, a fraction collector was used to collect 40 mL of the diluted reaction mixture containing a theoretical product amount of 0.976 mmol. Immediately after collection, the samples were poured into a suspension of sodium borohydride (148 mg, 3.9 mmol, 4 equiv) in 10 mL of ethanol at 0 °C. The vials were

rinsed with an additional 10 mL of ethanol and stirred for 30 min. 25 mL of saturated sodium bicarbonate was added and stirred, followed by 50 mL of water with further stirring. Any additional water was added to solubilize the precipitate prior to extraction. The reaction mixture was then transferred to a separatory funnel, the layers separated and the organic layer collected. The aqueous phase was extracted 2x with dichloromethane. The combined organic layers were dried over $MgSO_4$, filtered, and concentrated. The product was directly chromatographed with a gradient of 15% EtOAc/hexanes → 50% EtOAc/hexanes to afford the desired compound. The enantioselectivities were determined by chiral HPLC analysis.

Residence time (minutes)	Pump A flow rate (mL/min)	Pump B flow rate (mL/min)	Pump C or C+D flow rate (mL/min)
10	0.500	0.500	19.5
15	0.333	0.333	13.0
20	0.250	0.250	9.75
25	0.200	0.200	7.80

6. Assessing Long Term Stability and Activity of the Proline Packed Bed (Figure 4)

A glass Omnifit column (6.6 mm diameter) was packed with 1.00 g of L-proline and placed in the microreactor as described in the general setup. The column and tubing were flushed with ethyl acetate prior to use. A stock solution of nitrosobenzene (1 M) was prepared in ethyl acetate. A separate stock solution of thiourea **3b** (0.047 M) and hexanal (3 M) in ethyl acetate was also prepared. One stock solution was pumped from pump A and the other stock solution was pumped from pump B. The reactor was programmed to run 80 mL of aldehyde/thiourea stock solution through the microreactor and to periodically collect 20 mL fractions.

Immediately after collection, the desired samples were poured into a suspension of sodium borohydride (74 mg, 3.9 mmol, 4 equiv) in 5 mL of ethanol at 0 °C. The vials were rinsed with an additional 5 mL of ethanol and stirred for 30 min. 12.5 mL of saturated sodium bicarbonate was added and stirred, followed by 25 mL of water and further stirring. Any additional water was added to solubilize the precipitate prior to extraction. The reaction mixture was then transferred to a separatory funnel, the layers separated and the organic layer collected. The aqueous phase was extracted 2× with dichloromethane. The combined organic layers were dried over $MgSO_4$, filtered, and concentrated. The product was directly chromatographed with a gradient of 15% EtOAc/hexanes → 50% EtOAc/hexanes to afford the desired compound. Yields were plotted as a function of time in which the time is plotted as the midpoint of the sampling interval. The enantioselectivities were determined by chiral HPLC analysis.

Residence time (minutes)	Pump A flow rate (mL/min)	Pump B flow rate (mL/min)	Pump C flow rate (mL/min)
20	0.250	0.250	9.750

7. Representative Pump Trace – Table 2, entry 9

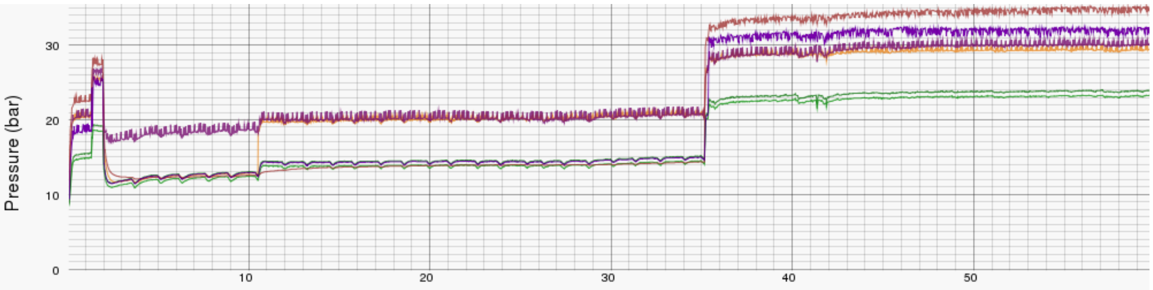

8. Product Characterization

General Reagent Configuration:
Reagents entering column (pump A): 3 M Aldehyde and 0.047 M **3b** in EtOAc
Reagents entering coil (pump B): 1 M nitrosobenzene in EtOAc
Reagents entering "quench" inlet: Ethyl Acetate

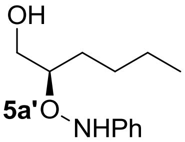

(*R*)-2-(*N*-phenylaminooxy)-hexan-1-ol (5a′). Prepared following the general reactor setup using hexanal (3.0 M) with a 0 °C packed bed L-proline column, 5 °C reactor coil, 20 min coil residence time (pump A = 0.250 mL/min, pump B = 0.250 mL/min, pump C = 9.750 mL/min), and a 1:1:39 (volumetric ratio for pump A: pump B: ethyl acetate dilution). 40 mL of product was collected (theoretical 0.976 mmol), reduced, and worked up following the procedure described in section 5. The product was purified by flash column chromatography on silica gel with 15% EtOAc/hexanes followed by 50% EtOAc/hexanes to afford the title compound as a yellow-orange oil (175.9 mg, 86% yield, 98% ee) ^{1}H NMR: (600 MHz, $CDCl_3$) δ 7.28 (m, 2H), 6.99 (m, 3H), 3.96 (m, 1H), 3.86 (dd, J = 2.5, 12.0 Hz, 1H), 3.78 (dd, J = 6.4, 12.1 Hz 1H), 2.58 (br s, 1H), 1.69 (m, 1H), 1.53 (m, 1H), 1.48–1.28 (m, 4H), 0.92 (t, J = 7.1 Hz, 3H) ppm. ^{13}C NMR (151 MHz, $CDCl_3$) δ 148.7, 129.1, 122.4, 114.9, 84.1, 65.2, 29.8, 28.0, 23.0, 14.1 ppm.

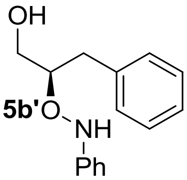

(*R*)-3-phenyl-2-(*N*-phenylaminooxy)-propan-1-ol (5b′). Prepared according to the general reactor setup using hydrocinnamaldehyde (3.0 M) with a 0 °C packed bed L-proline column, 5 °C reactor coil, 10 minute coil residence time (pump A = 0.500 mL/min, pump B = 0.500 mL/min, pump C = 9.750 mL/min) and a 1:1:19.5 (volumetric ratio for pump A/pump B/ethyl acetate dilution). 20.98 ml of diluted product was collected (theoretical 0.976 mmol), reduced, and worked up following the procedure

described in section 5. The product was purified by flash column chromatography on silica gel with 20% diethyl ether/pentane followed by 50% diethyl ether/pentane) to afford the title compound as a yellow oil (179.3 mg, 76% yield, 99% ee). ^{1}H NMR: (600 MHz, $CDCl_3$) δ 7.34–7.15 (m, 7H), 7.04 (br s, 1H), 6.95 (m, 1H), 6.85 (m, 2H), 4.16 (m, 1H), 3.86 (dd, J = 2.4, 12.0 Hz,1H), 3.74 (dd, J = 5.9, 12.1 Hz, 1H), 3.06 (dd, J = 6.8, 13.7 Hz, 1H), 2.86 (dd, J = 7.1, 13.8 Hz, 1H), 2.38 (br s, 1H) ppm. ^{13}C NMR (151 MHz, $CDCl_3$) δ 148.5, 138.1, 129.6, 129.1, 128.6, 126.6, 122.5, 114.8, 85.2, 64.2, 36.6 ppm.

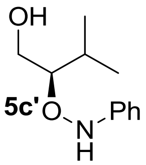

(*R*)-3-methyl-2-(*N*-phenylaminooxy)-butan-1-ol (5c′). Prepared according to the general reactor setup using isovaleraldehyde (3.0 M) with a 40 °C packed bed L-proline column, 20 °C reactor coil, 20 minute coil residence time (pump A = 0.200 mL/min, pump B = 0.200 mL/min, and pump C = 7.8 mL/min) and a 1:1:39 (volumetric ratio for pump A/pump B: ethyl acetate dilution). 40 mL of diluted product was collected (theoretical 0.976 mmol), reduced and worked up following the general procedure described in section 5. The product was purified by flash column chromatography on silica gel with 15% EtOAc/hexanes followed by 50% EtOAc/hexanes to afford the title compound as a yellow-orange oil (146.6 mg, 77% yield, 97% ee). ^{1}H NMR: (600 MHz, $CDCl_3$) δ 7.27 (m, 2H), 7.07 (br s, 1H), 7.03–6.96 (m, 3H), 3.87 (m, 2H), 3.74 (td, J = 6.0, 3.0 Hz, 1H), 2.79 (br s, 1H), 2.03 (m, 1H), 1.05 (d, J = 6.9 Hz, 3H), 1.00 (d, J = 6.9 Hz, 3H) ppm. ^{13}C NMR (151 MHz, $CDCl_3$) δ 148.5, 129.2, 122.8, 115.3, 88.9, 64.0, 29.0, 19.0, 18.8 ppm.

9. References

1. Giera, H.; Meiers, M.; Hugger, U., Process for the Preparation of nitrosobenzenes, U.S. Patent 6,274,775, August 14, 2001.

10. Chromatograms of Racemic and Enantiomerically Enriched Products

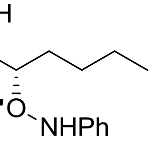

5a'

Method: CHIRALPAK IA and guard column 95% hex 5% ipa, 1.00 ml/min, 254 nm

Racemic

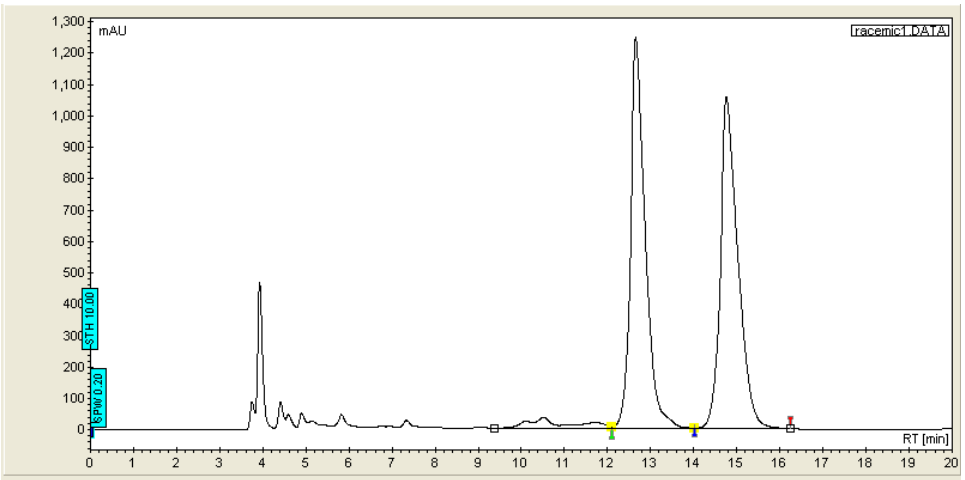

#	Name	Time [Min]	Quantity [% Area]	Height [mAU]	Area [mAU.Min]	Area % [%]
1	UNKNOWN	12.65	49.60	1244.5	489.5	49.597
2	UNKNOWN	14.76	50.40	1054.6	497.4	50.403
Total			100.00	2299.1	986.9	100.000

Enantioenriched – 99% ee

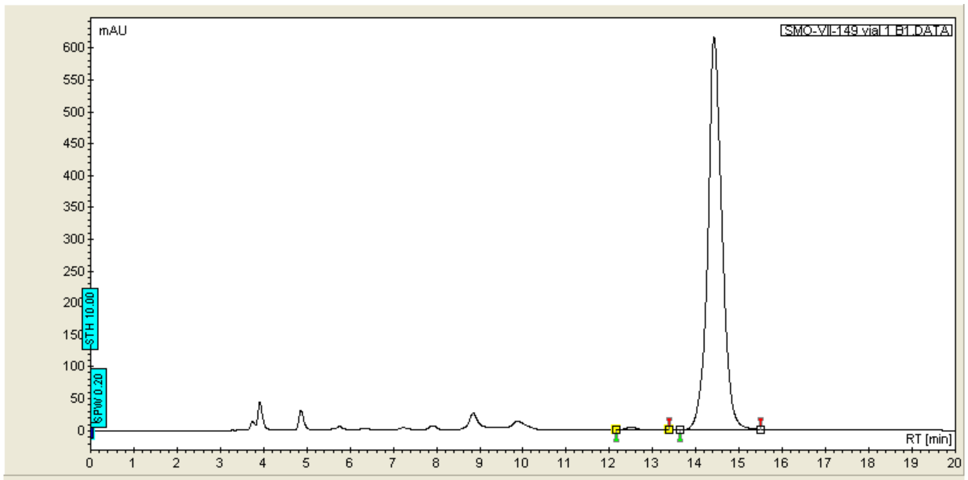

#	Name	Time [Min]	Quantity [% Area]	Height [mAU]	Area [mAU.Min]	Area % [%]
1	UNKNOWN	12.50	0.68	4.9	1.6	0.678
2	UNKNOWN	14.42	99.32	615.7	234.4	99.322
Total			100.00	620.6	236.0	100.000

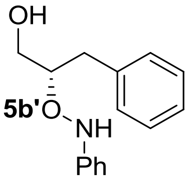

Method: CHIRALPAK IA and guard column 95% hex 5% ipa, 1.00 ml/min, 254 nm
Racemic

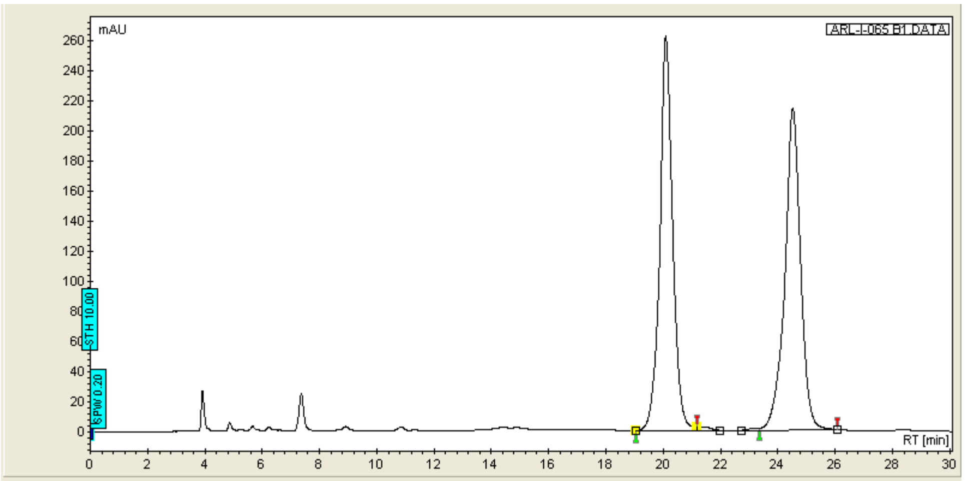

#	Name	Time [Min]	Quantity [% Area]	Height [mAU]	Area [mAU.Min]	Area % [%]
1	UNKNOWN	20.09	49.81	262.3	135.7	49.813
2	UNKNOWN	24.53	50.19	214.3	136.7	50.187
Total			100.00	476.6	272.3	100.000

Enantioenriched – 99% ee

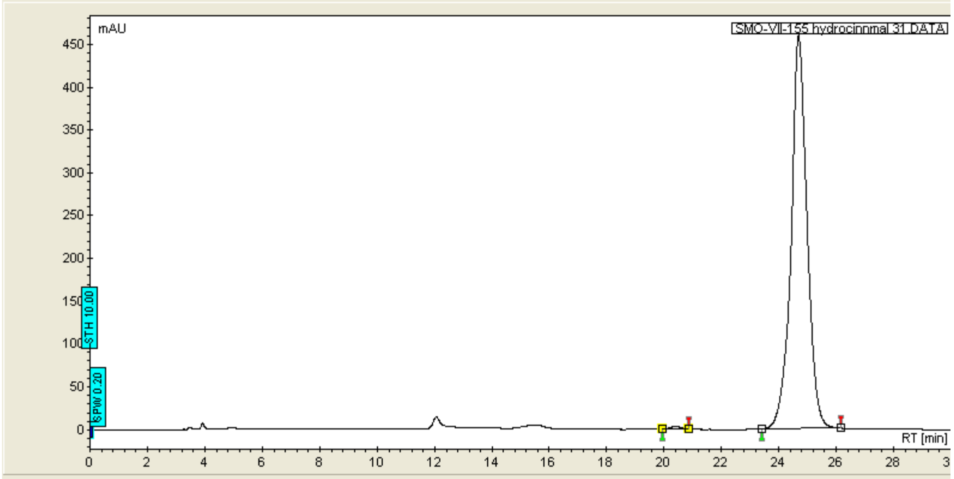

#	Name	Time [Min]	Quantity [% Area]	Height [mAU]	Area [mAU.Min]	Area % [%]
1	UNKNOWN	20.41	0.44	3.1	1.3	0.436
2	UNKNOWN	24.69	99.56	459.5	296.0	99.564
Total			100.00	462.6	297.3	100.000

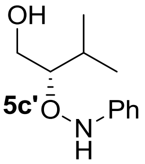

5c'

Method: CHIRALPAK IA and guard column 95% hex 5% ipa, 1.00 ml/min, 254 nm

Racemic

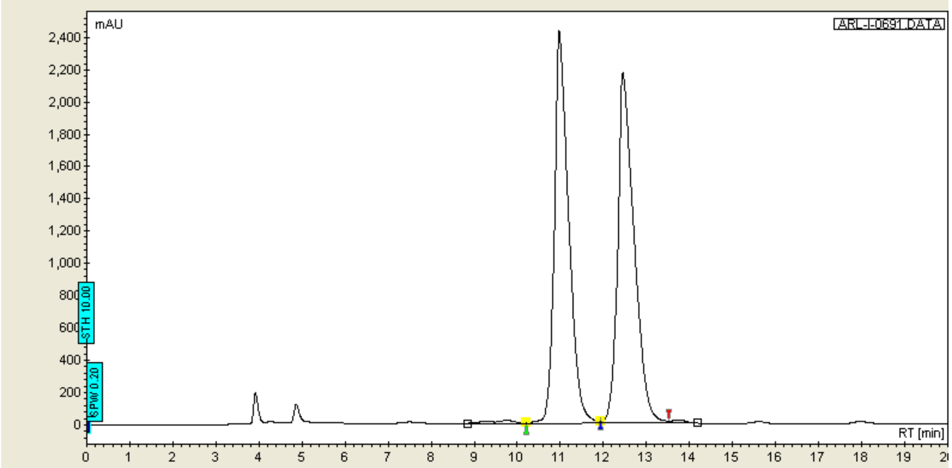

#	Name	Time [Min]	Quantity [% Area]	Height [mAU]	Area [mAU.Min]	Area % [%]
1	UNKNOWN	10.97	49.25	2432.7	931.7	49.254
2	UNKNOWN	12.45	50.75	2172.2	959.9	50.746
Total			100.00	4604.9	1891.7	100.000

Enantioenriched – 97% ee

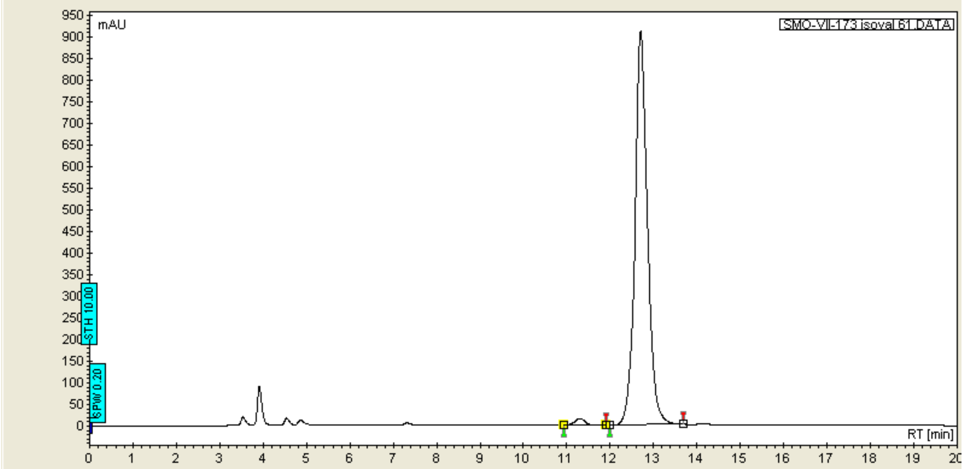

#	Name	Time [Min]	Quantity [% Area]	Height [mAU]	Area [mAU.Min]	Area % [%]
1	UNKNOWN	11.31	1.36	15.4	4.2	1.359
2	UNKNOWN	12.70	98.64	912.1	303.8	98.641
Total			100.00	927.4	308.0	100.000

11. ^{1}H and ^{13}C NMR Spectra of Chemical Compounds

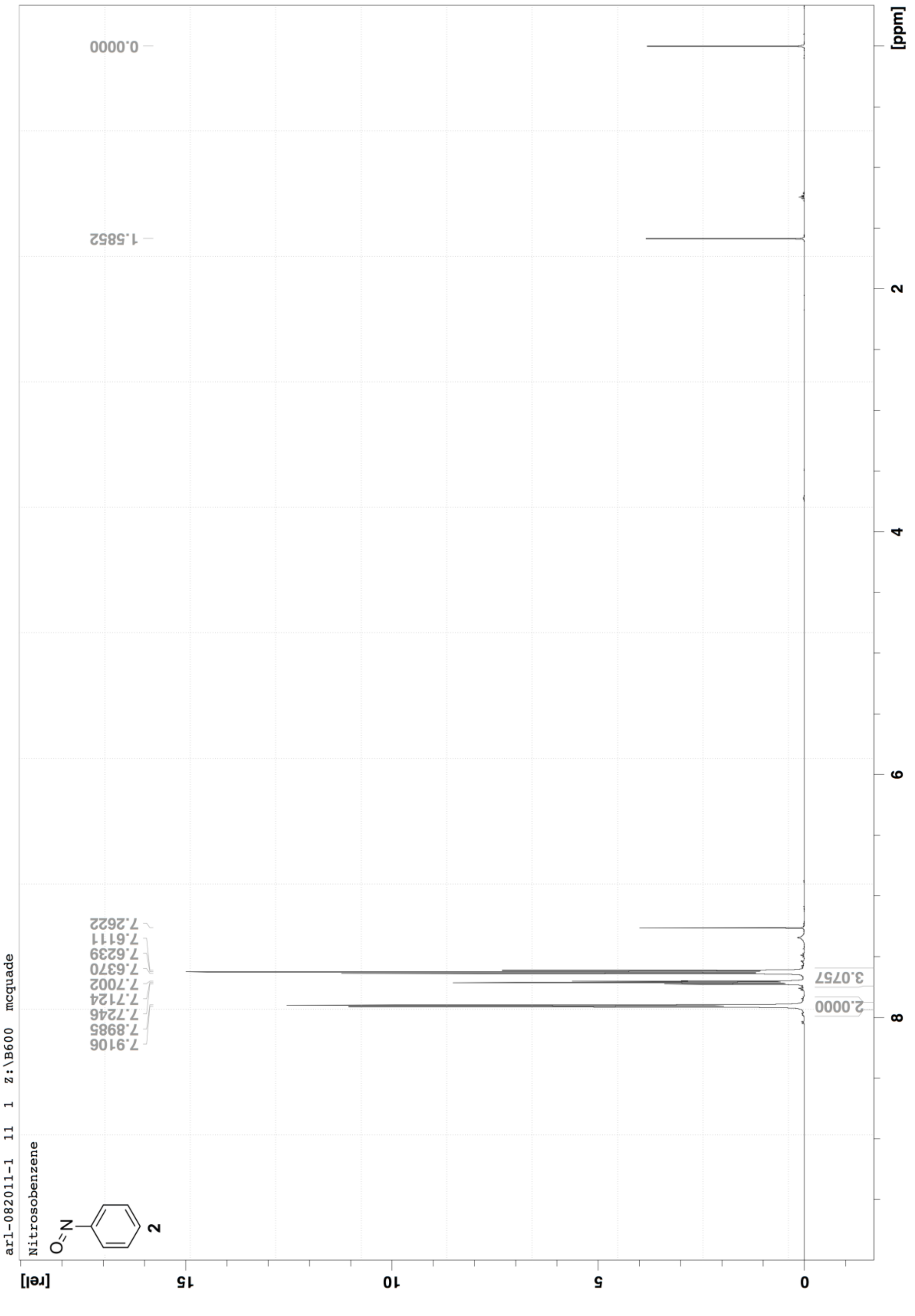

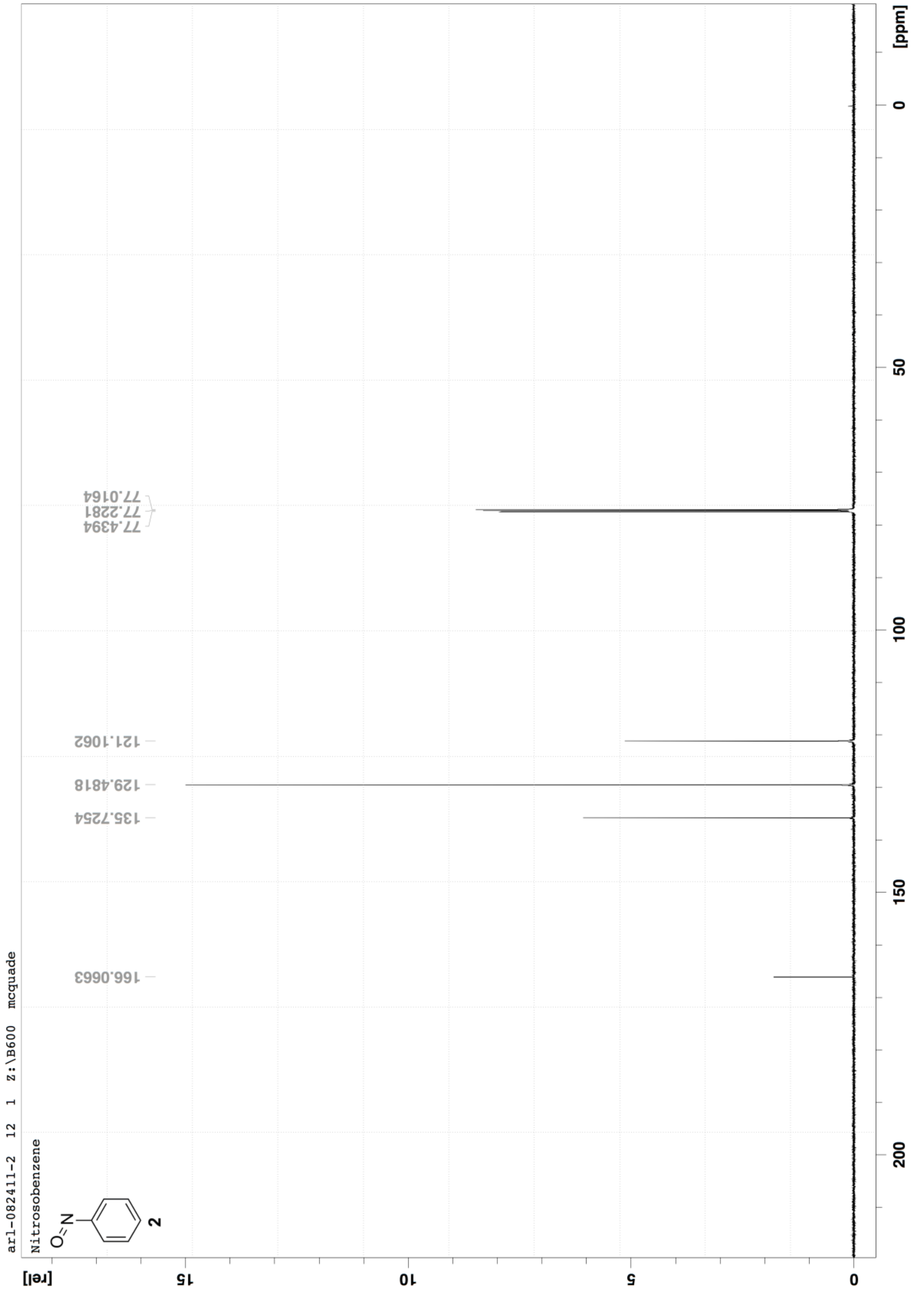
arl-082411-2 12 1 Z:\B600 mcquade
Nitrosobenzene
2
166.0663
135.7254
129.4818
121.1062
77.4394
77.2281
77.0164
[rel]
[ppm]

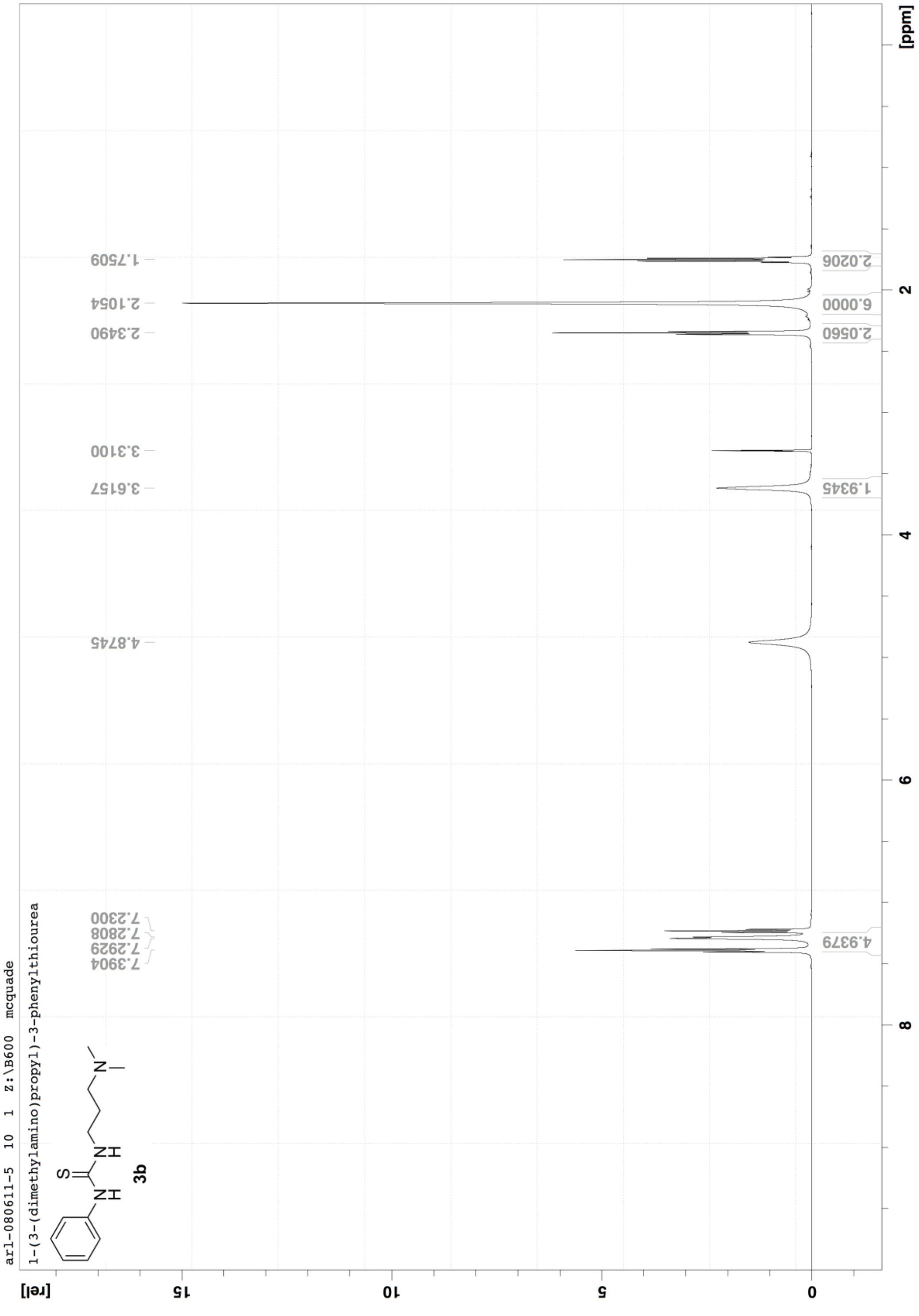
arl-080611-5 10 1 Z:\B600 mcquade
1-(3-(dimethylamino)propyl)-3-phenylthiourea
3b
7.3904
7.2929
7.2808
7.2300
4.8745
3.6157
3.3100
2.3490
2.1054
1.7509
4.9379
1.9345
2.0560
6.0000
2.0206
[ppm]
[rel]

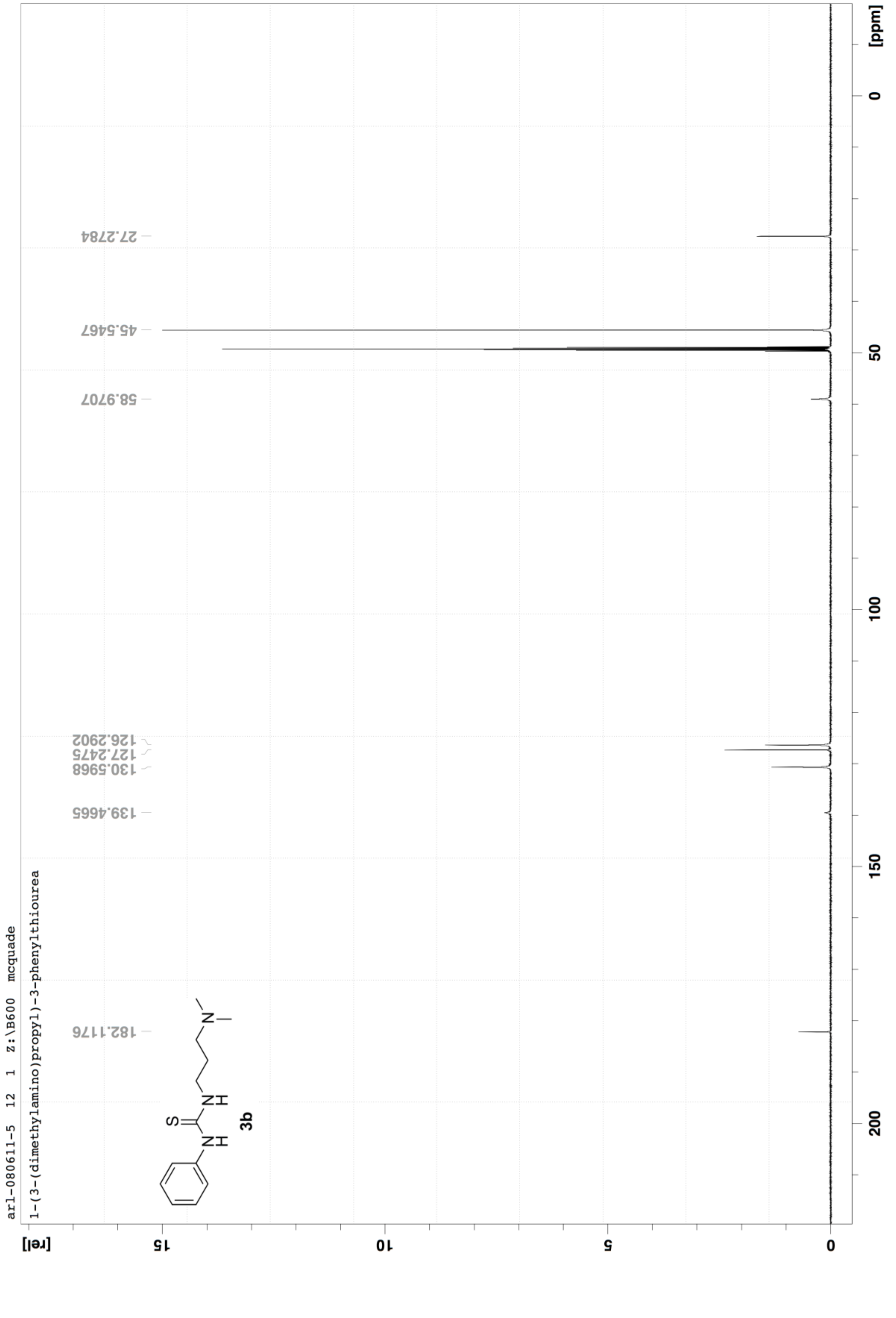
arl-080611-5 12 1 Z:\B600 mcquade
1-(3-(dimethylamino)propyl)-3-phenylthiourea
3b
182.1176
139.4665
130.5968
127.2475
126.2902
58.9707
45.5467
27.2784
[ppm]
[rel]

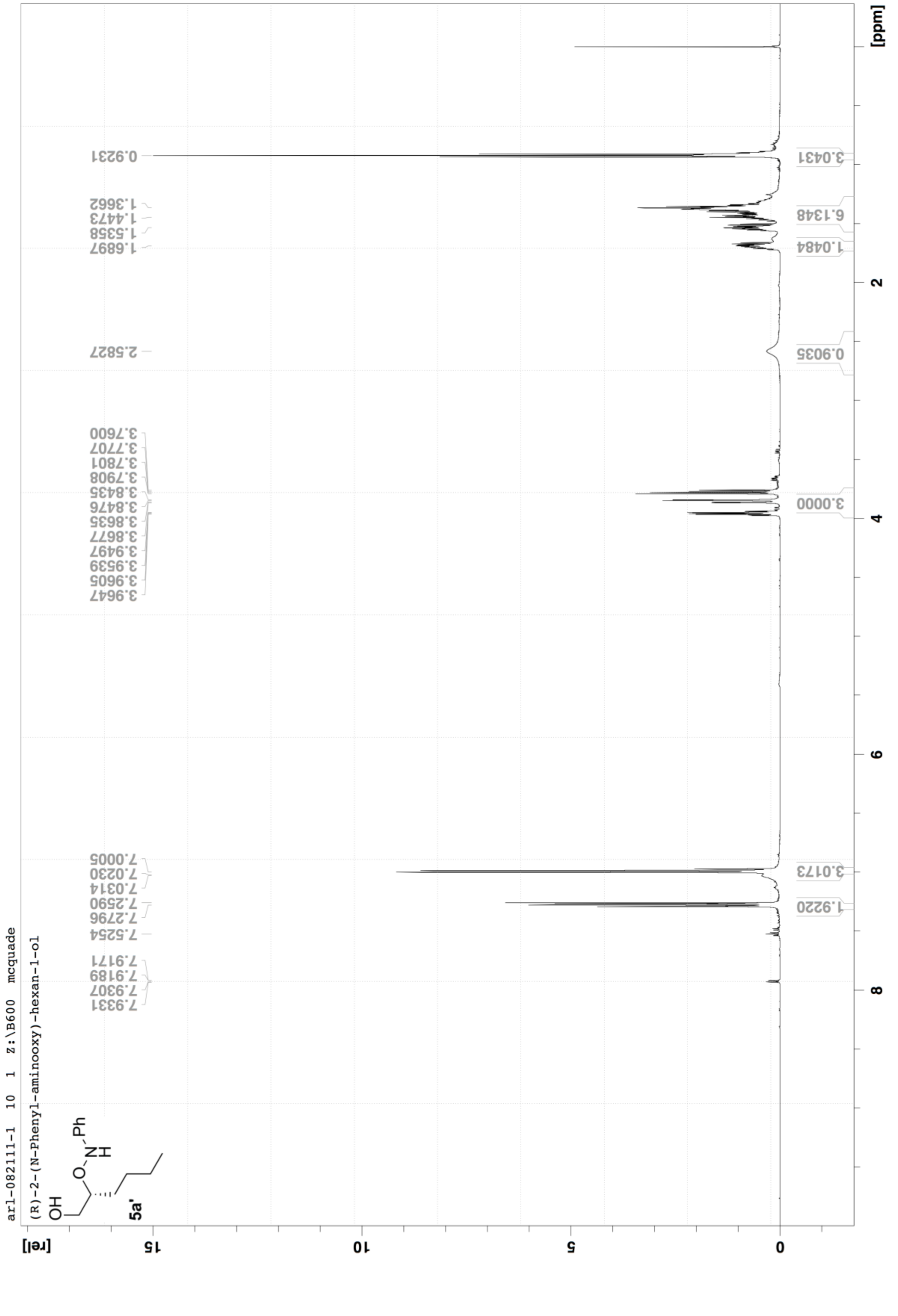
arl-082111-1 10 1 Z:\B600 mcquade
(R)-2-(N-Phenyl-aminooxy)-hexan-1-ol
OH
O
N
H
Ph
5a'
7.9331
7.9307
7.9189
7.9171
7.5254
7.2796
7.2590
7.0314
7.0230
7.0005
3.9647
3.9605
3.9539
3.9497
3.8677
3.8635
3.8476
3.8435
3.7908
3.7801
3.7707
3.7600
2.5827
1.6897
1.5358
1.4473
1.3662
0.9231
1.9220
3.0173
3.0000
0.9035
1.0484
6.1348
3.0431
[rel]
15
10
5
0
8
6
4
2
[ppm]

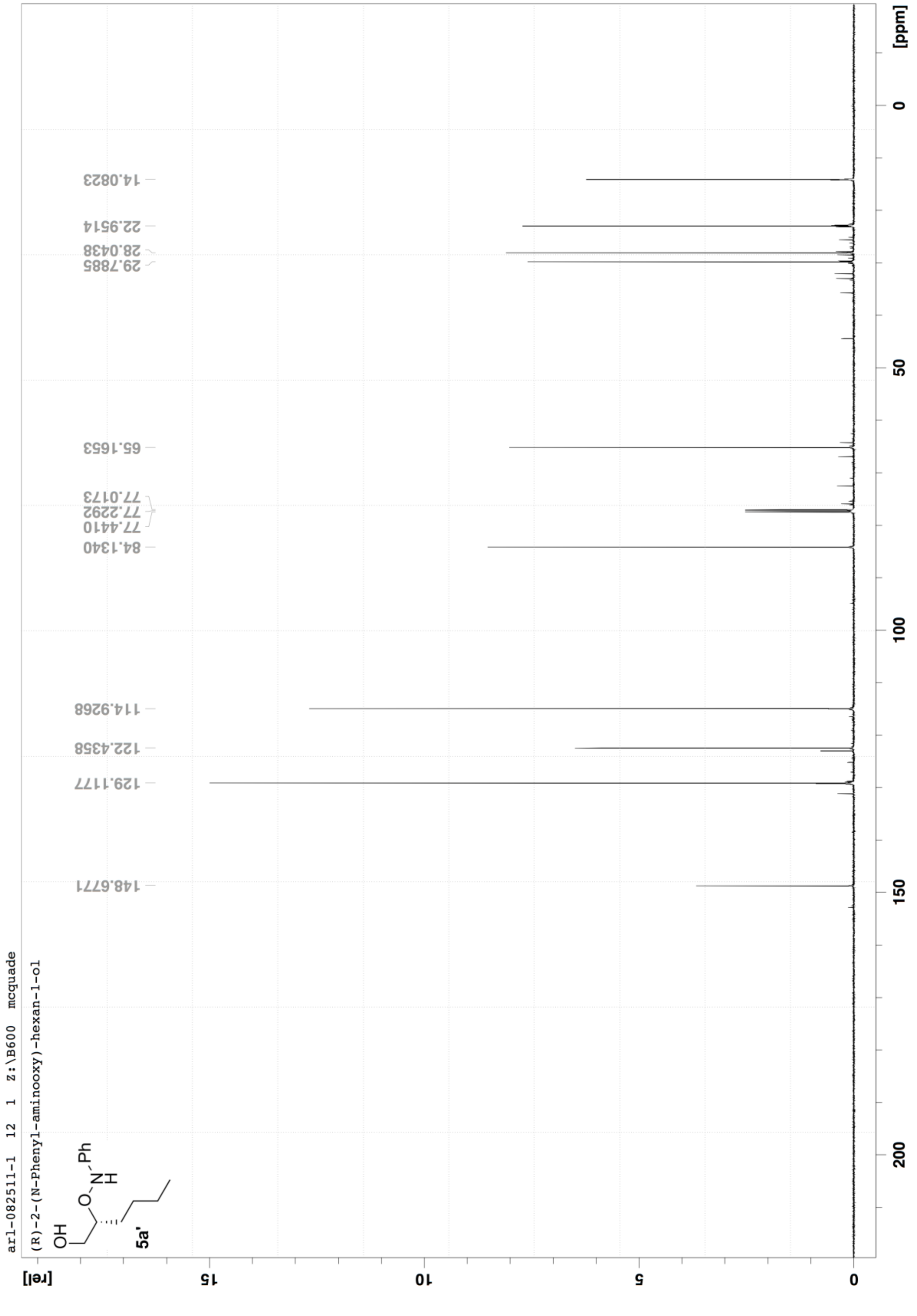

arl-082511-1 12 1 Z:\B600 mcquade
(R)-2-(N-Phenyl-aminooxy)-hexan-1-ol
5a'
148.6771
129.1177
122.4358
114.9268
84.1340
77.4410
77.2292
77.0173
65.1653
29.7885
28.0438
22.9514
14.0823
[ppm]
[rel]

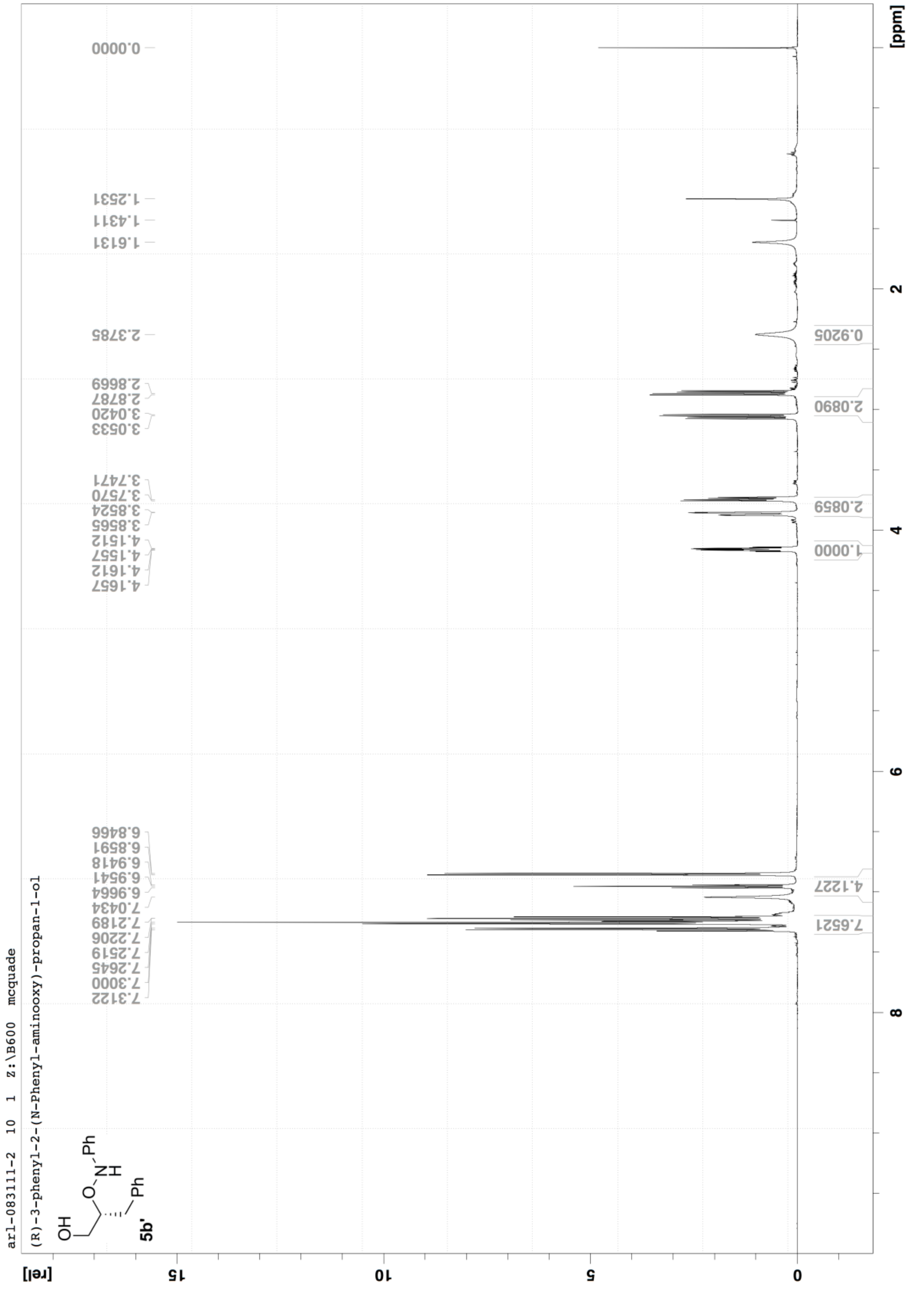
arl-083111-2 10 1 Z:\B600 mcquade
(R)-3-phenyl-2-(N-Phenyl-aminooxy)-propan-1-ol
5b'
[ppm]
[rel]

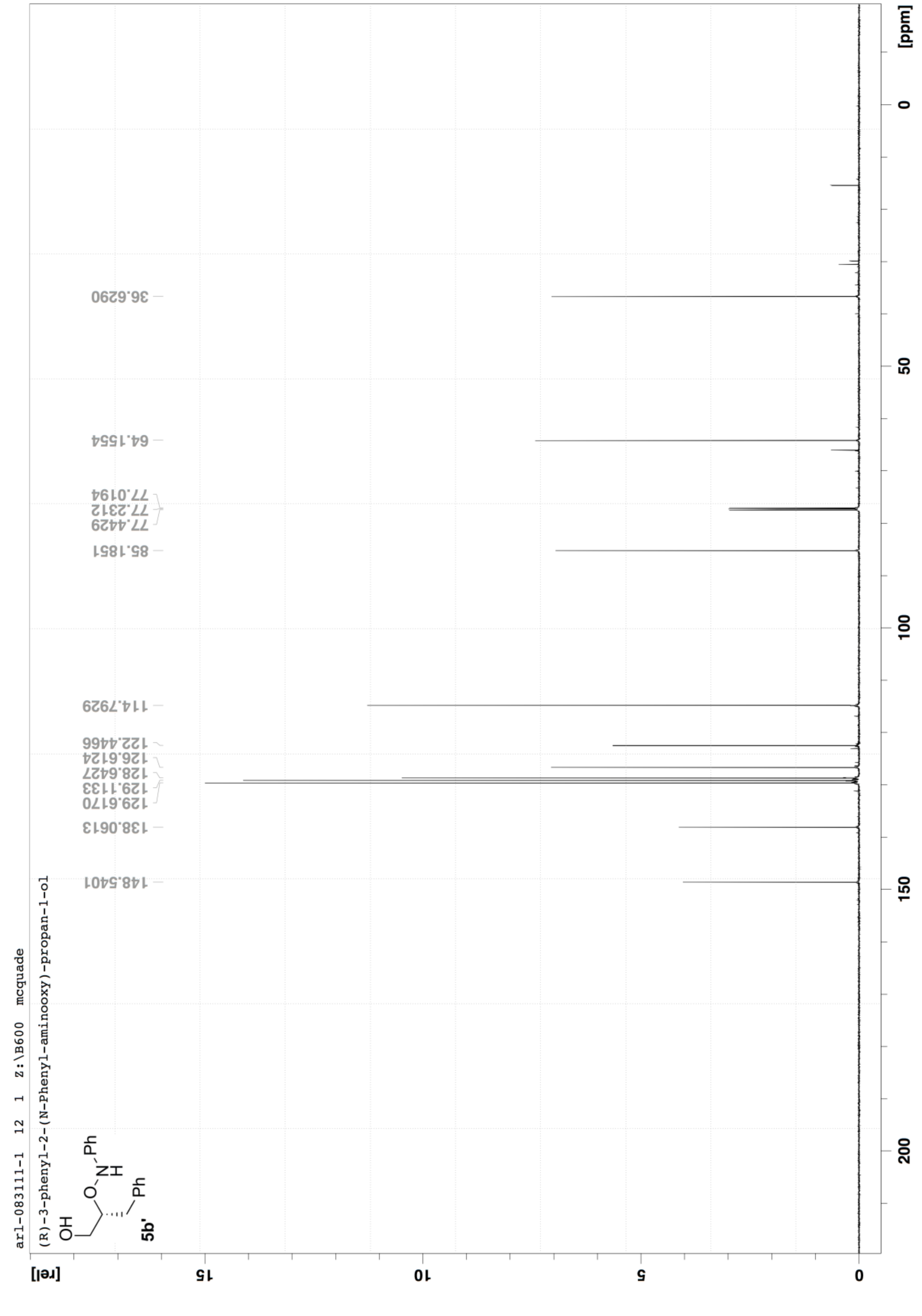
arl-083111-1 12 1 Z:\B600 mcquade
(R)-3-phenyl-2-(N-Phenyl-amninooxy)-propan-1-ol
5b'
148.5401
138.0613
129.6170
129.1133
128.6427
126.6124
122.4466
114.7929
85.1851
77.4429
77.2312
77.0194
64.1554
36.6290
[ppm]
[rel]

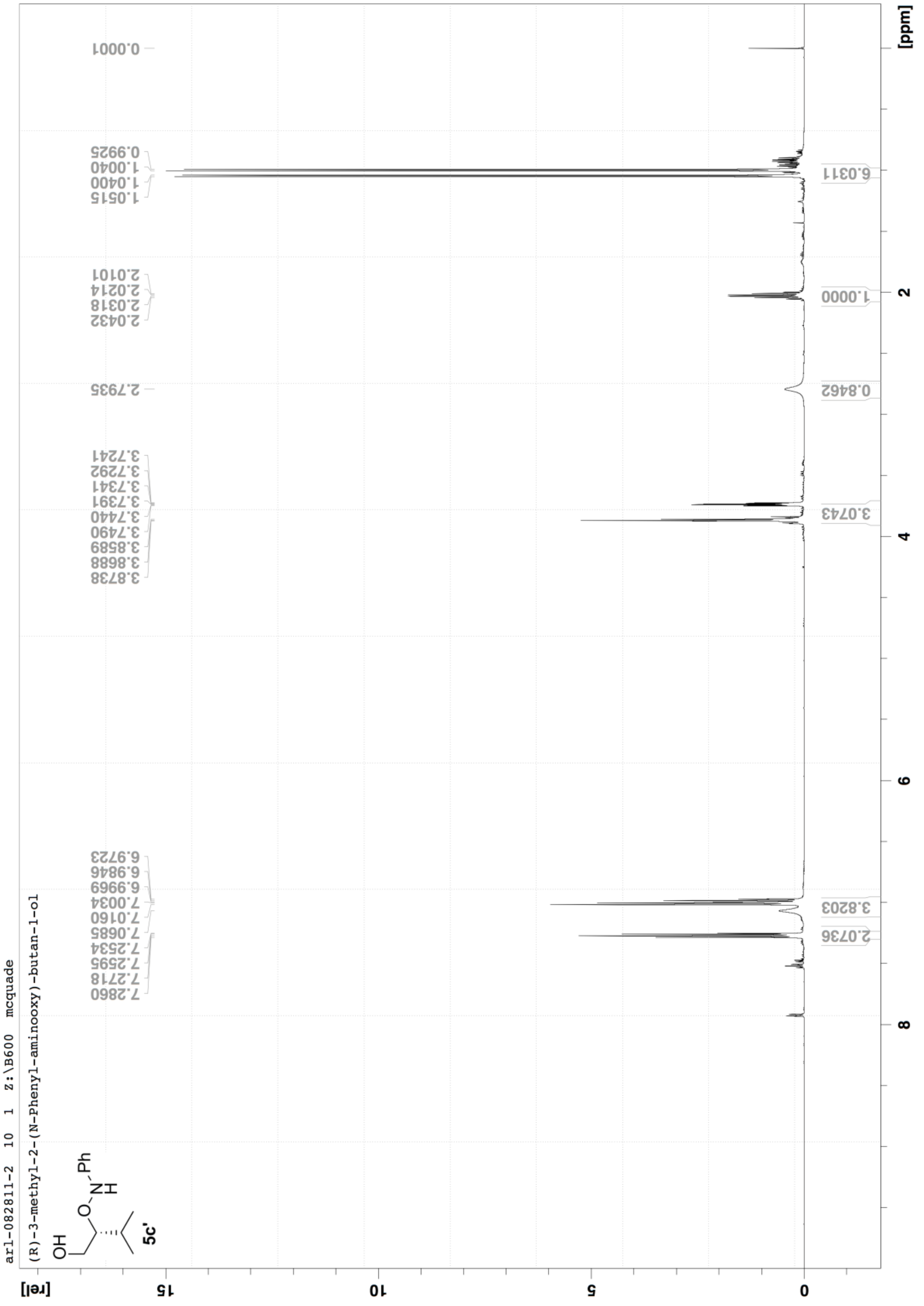
arl-082811-2 10 1 Z:\B600 mcquade
(R)-3-methyl-2-(N-Phenyl-aminooxy)-butan-1-ol
OH
O
N
H
Ph
5c'
7.2860
7.2718
7.2595
7.2534
7.0685
7.0160
7.0034
6.9969
6.9846
6.9723
3.8738
3.8688
3.8589
3.7490
3.7440
3.7391
3.7341
3.7292
3.7241
2.7935
2.0432
2.0318
2.0214
2.0101
1.0515
1.0400
1.0040
0.9925
0.0001
2.0736
3.8203
3.0743
0.8462
1.0000
6.0311
[ppm]
[rel]

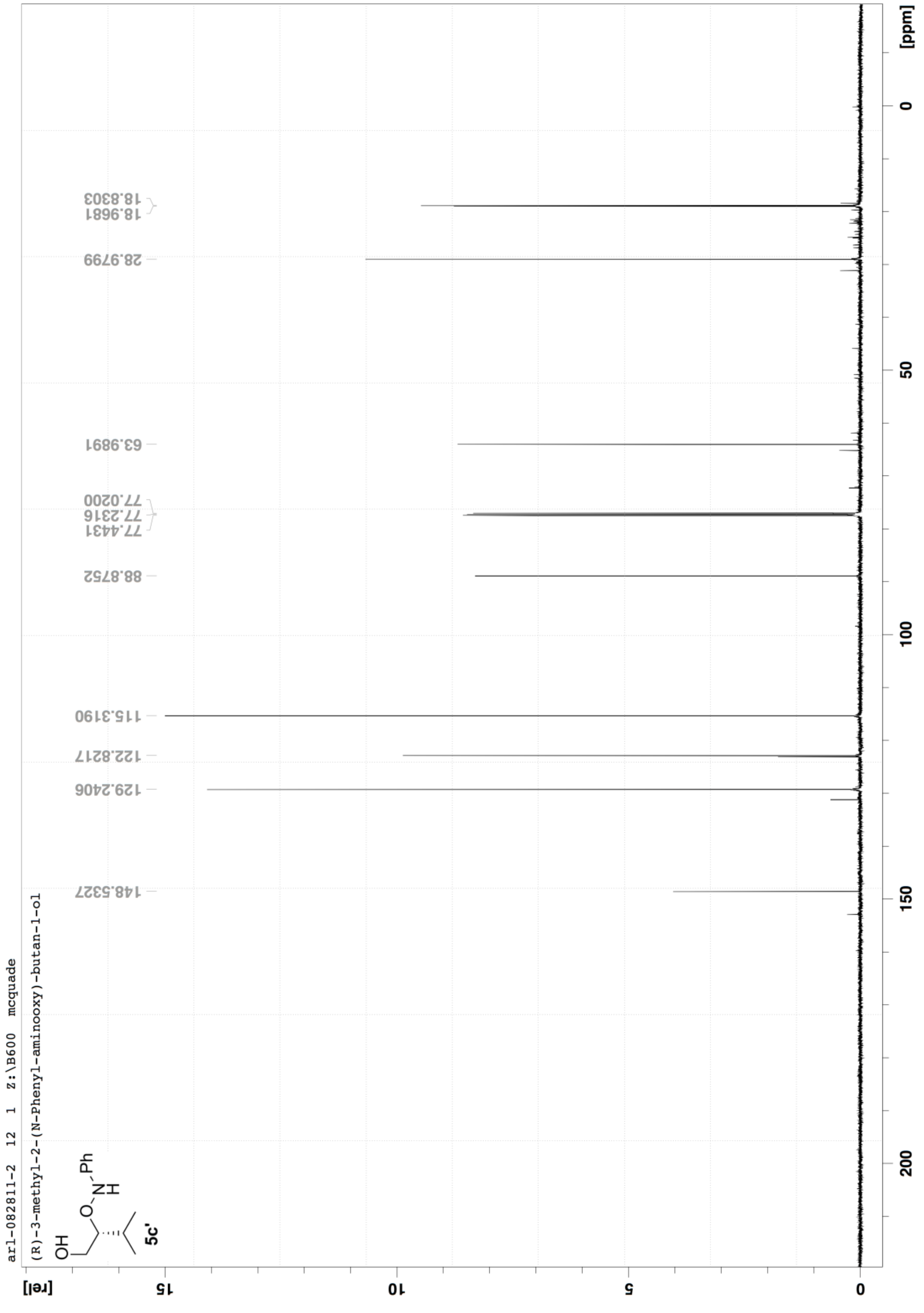
arl-082811-2 12 1 Z:\B600 mcquade
(R)-3-methyl-2-(N-Phenyl-aminooxy)-butan-1-ol
5c'
148.5327
129.2406
122.8217
115.3190
88.8752
77.4431
77.2316
77.0200
63.9891
28.9799
18.9681
18.8303
[ppm]
[rel]

Crude reaction mixture for **3b** alone going through the L-proline packed bed column

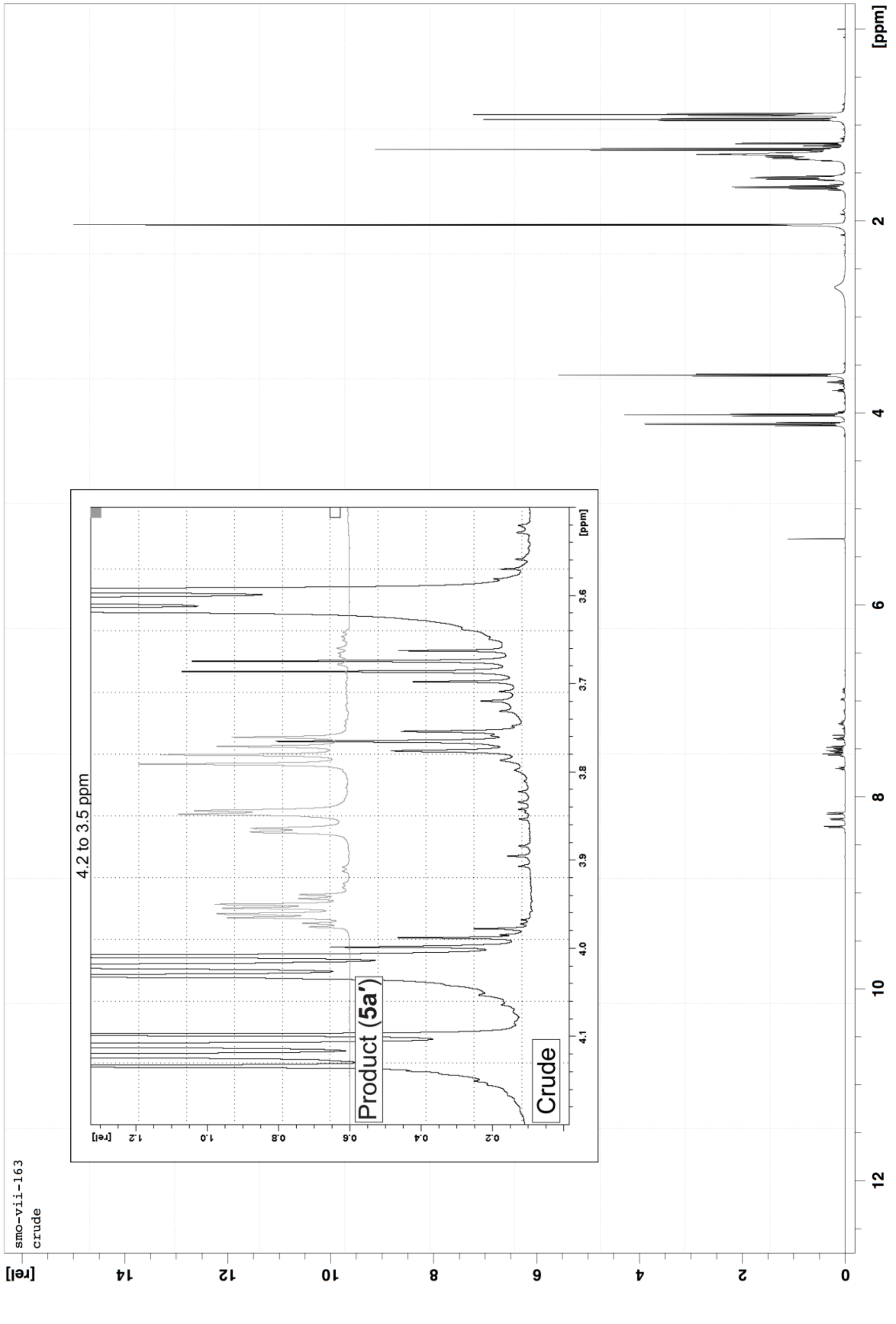

Crude reaction mixture with no **3b** present

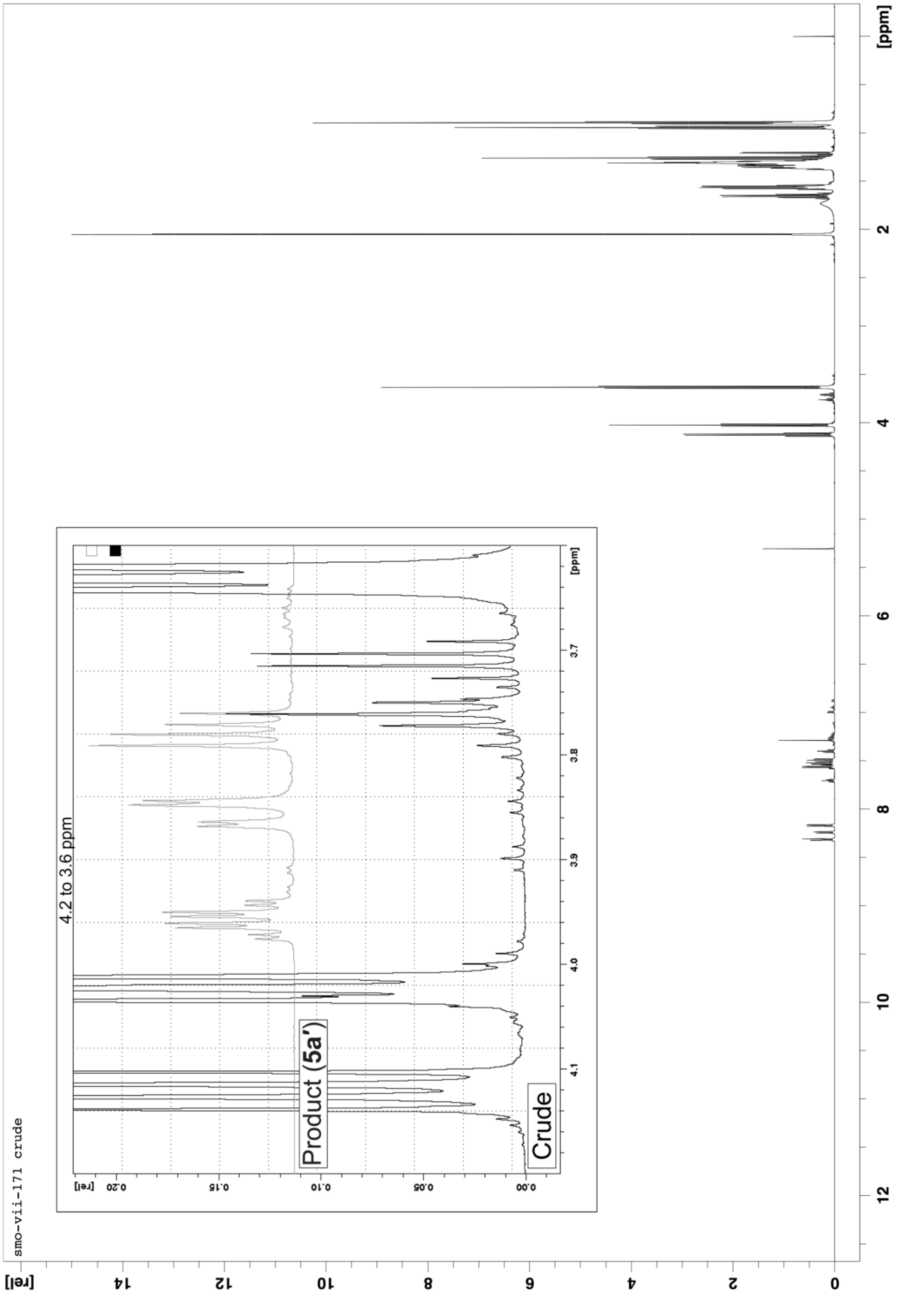

Supplement: File 1 — Experimental Section. [file Beilstein_J_Org_Chem-07-1671-s001.pdf]
